# Diversification of insects since the Devonian: a new approach based on morphological disparity of mouthparts

**DOI:** 10.1038/s41598-018-21938-1

**Published:** 2018-02-23

**Authors:** Patricia Nel, Sylvain Bertrand, André Nel

**Affiliations:** 1 Institut de Systématique, Évolution, Biodiversité, ISYEB-UMR 7205-CNRS, MNHN, UPMC, EPHE, Muséum national d’Histoire naturelle, Sorbonne Universités, 57 rue Cuvier, CP 50, Entomologie, F-75005 Paris, France; 2 AgroParisTech, 75005 Paris, France

## Abstract

The majority of the analyses of the evolutionary history of the megadiverse class Insecta are based on the documented taxonomic palaeobiodiversity. A different approach, poorly investigated, is to focus on morphological disparity, linked to changes in the organisms’ functioning. Here we establish a hierarchy of the great geological epochs based on a new method using Wagner parsimony and a ‘presence/absence of a morphological type of mouthpart of Hexapoda’ dataset. We showed the absence of major rupture in the evolution of the mouthparts, but six epochs during which numerous innovations and few extinctions happened, i.e., Late Carboniferous, Middle and Late Triassic, ‘Callovian-Oxfordian’, ‘Early’ Cretaceous, and ‘Albian-Cenomanian’. The three crises Permian-Triassic, Triassic-Jurassic, and Cretaceous-Cenozoic had no strong, visible impact on mouthparts types. We particularly emphasize the origination of mouthparts linked to nectarivory during the Cretaceous Terrestrial Revolution. We also underline the origination of mouthparts linked to phytophagy during the Middle and the Late Triassic, correlated to the diversification of the gymnosperms, especially in relation to the complex ‘flowers’ producing nectar of the Bennettitales and Gnetales.

## Introduction

During their ca. 410 Ma history, hexapods have evolved morphologically to adapt in a continuously changing world, thereby resulting in a unique mega-biodiversity^1^. Age-old questions^2–4^ about insects’ macroevolution nowadays receive renewed interest thanks to the remarkable recent improvements in data and methods that allow incorporating full data, phylogenomic trees besides fossil record^5–9^. Also significant advances in knowledge on fossil entomofaunas took place during the last 15 years, due to discoveries and studies of several rich sites with exceptional preservation, as well to changes in taxonomy and dating of fossil deposits, rendering useful updates of the previous works.

The insect evolutionary history remains mainly analyzed on the basis of the documented families and orders, ignoring two inherent weaknesses^2–15^: the extensions and definitions of the fossil and recent families vary frequently depending on the authors, and the monophyly of fossil groups of high rank is difficult to establish (e.g., the ‘Grylloblattodea’, ‘Protorthoptera’, Meganisoptera, etc.)^16,17^ (for discussion see^18^, Supporting Information).

A different approach to study the past evolution of insects in a palaeoecological perspective emphasizes on morphological disparity rather than on taxonomic diversity. The first studies of this kind on insects concerned the mouthparts^11,19^, analyzing the evolution of ‘feeding guilds’ particularly in relation to the angiosperms’ diversification. These guilds were based on mouthpart classes recorded as clusters using distance and agglomeration from a matrix of presence/absence of types of mouthparts^10,19^.

Here we also propose to restudy the insect macroevolution based on mouthpart disparity instead of diversification of taxa, but by using a newly defined set of characters and a protocol of analysis different from the one used previously. We will not cluster mouthpart types into classes because it can result in artificially putting together phylogenetically distant insects^20^, with a risk of not translating correctly analogous but non homologous adaptations.

Our analysis is based on the recently developed method, WAPUM, ‘Wagner Parsimony Analysis Applied to Palaeosynecology using Morphology’^18,21^, to establish a hierarchy (‘tree’) of the geological epochs based on the appearances/extinctions of types of insect mouthparts through time. The Wagner parsimony method searches the hierarchies (nested sets of objects) that are best compatible with the distribution of character states among the objects to classify. Unlike its application in the cladistic method, this method is independent of all ideas of phylogenetic sequences and is currently used in synecology to classify landscape parts. It is also used in palaeosynecology to classify geological periods or palaeontological localities (for review see^18^). Here we hierarchize the currently accepted geological epochs^22^. Each corresponds to time period delimited by more or less important extinction events, resulting or due to changes in the ecosystems^23^. We also define two ‘artificial’ ones grouping the Callovian with the Oxfordian (for a Middle-Late Jurassic) and the Albian with the Cenomanian because the main outcrops with insects for these periods are straddling these epochs (Supporting Information). In WAPUM the characters are based on morphology and the method has already successfully been tested for particular insect clades (Odonatoptera, Thripida, Dermaptera)^18,21^. We define the characters as follows: ‘presence vs. absence of taxa bearing a particular mouthpart type’. The mouthpart types are defined in the Supporting Information.

This approach based on morphological characters presents the great advantage not being biased by paraphyly or polyphyly. We privilege the Wagner Parsimony method rather than phenetic ones to avoid as many *a priori* assumptions as possible in the choices of the distance and agglomeration tools. A second advantage is that the method is falsifiable as is the cladistics approach in phylogeny^24^, by additions of new characters to the matrix and comparison to attributes. For methods, tools, and their limits see Supporting Information. Analyses of the obtained hierarchies concern their topologies, ‘gradual’ or not, the ‘rakes’ vs. complete resolutions, and the number and nature of the mouthpart types supporting the transitions between epochs.

## Results and Discussion

**Figure 1:**
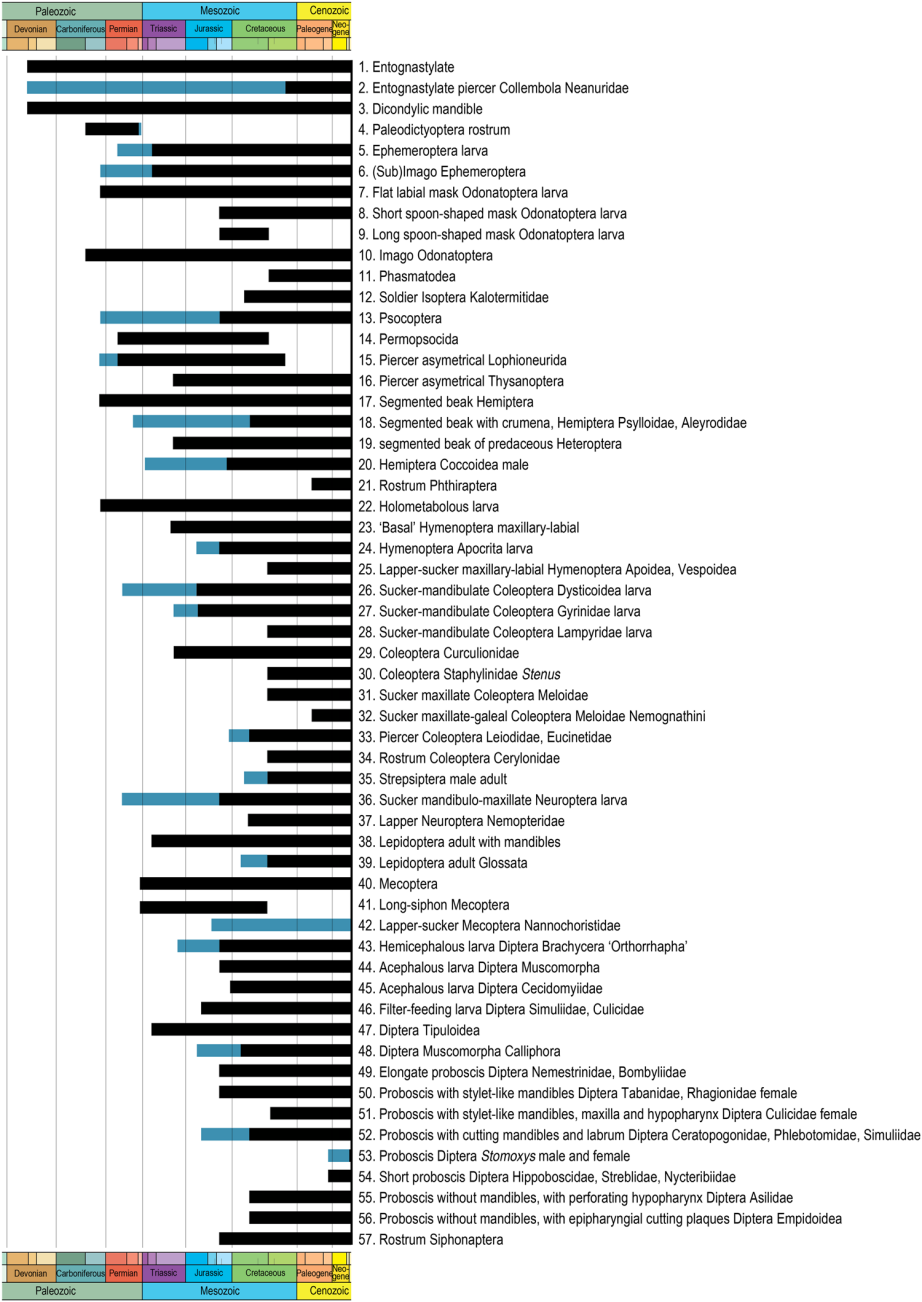
List of the 57 mouthpart types. Short descriptions and histogram of fossil occurrences. In black presence of a fossil bearing the morphological mouthpart type or belonging to the crown group bearing it (corresponding character state ‘1’ in Matrix Table S1). In blue presence of a fossil inside stem group with mouthparts not preserved (corresponding character state ‘?’ in Matrix Table S1). The shortest vertical lines inside Jurassic and Cretaceous refer to the subdivisions ‘Callovian-Oxfordian’ and ‘Albian-Cenomanian’. Copyright P.N.

We identified 57 types of mouthparts and reported their distribution during time (Fig. 1, Supporting Information). Some types concern orders, groups of families, while other are found in single subfamilies. The chosen types are characterized by their high specialization in feeding and biology, and/or important modifications of the structures.

### Topology of the hierarchy

**Figure 2:**
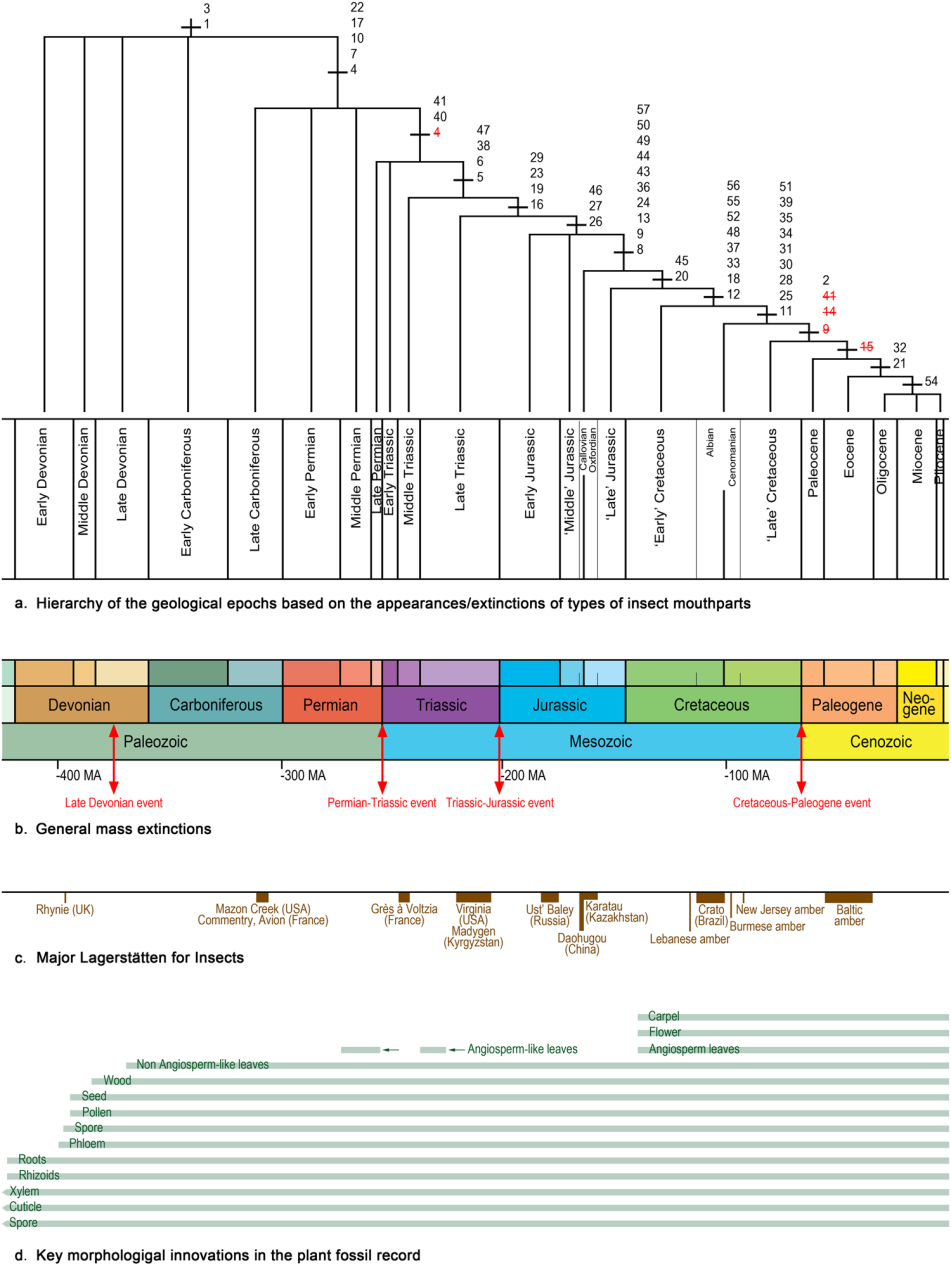
Results (**a**) Hierarchy of the geological epochs based on the appearances/extinctions of types of insect mouthparts through time using ‘Wagner Parsimony Analysis Applied to Palaeosynecology using Morphology’^18,21^; numbers are those assigned to the mouthparts types supporting the hierarchy, in black first records, in red and strikethrough extinctions. (**b**) Dates of main mass extinctions, recorded for marine organisms, time scale based on currently accepted geological epochs^22^ plus two ‘artificial’ ones: ‘Callovian-Oxfordian’ (for a Middle-Late Jurassic) and ‘Albian-Cenomanian’. (**c**) Positions and approximate durations of major insect Lagerstätten. (**d**) Key morphological innovations in plant fossil record, partly based on^53^. (**d**) is not covered by the CC BY licence. [EVOLUTION OF PLANTS 2E by K. J. Willis & J. C. McElwain (2013): Figure 9.4 (p.304)], all rights reserved, used with permission from Oxford University Press. Copyright P.N. for other figures.

The consensus hierarchy shows five non-resolutions or ‘rakes’, related to impossibilities for the software to distinguish the concerned epochs on the basis of ‘apomorphies’. The reasons for that are of two types: three of these situations are attributable to epochs of relative lack of fossil deposits, i.e. the ‘arthropod gap’ of the Late Devonian - Early Carboniferous (for the rake with the Middle and Late Devonian plus the Early Carboniferous), or to the lack of rich outcrops in the Early Triassic (for the rake with the Late Permian and Early Triassic), and the relative lack of rich Lagerstätten in the Early Jurassic compared to the Late Triassic (for the rake with the Early Jurassic and ‘Middle’ Jurassic) (Fig. 2c). The other two ‘rakes’, concerning the Late Carboniferous to the Middle Permian and the Oligocene to the Pliocene (a period with modern entomofauna), correspond to very few significant changes in the mouthpart morphologies, while these periods correspond to relatively rich fossil records.

### Originations

**Figure 3:**
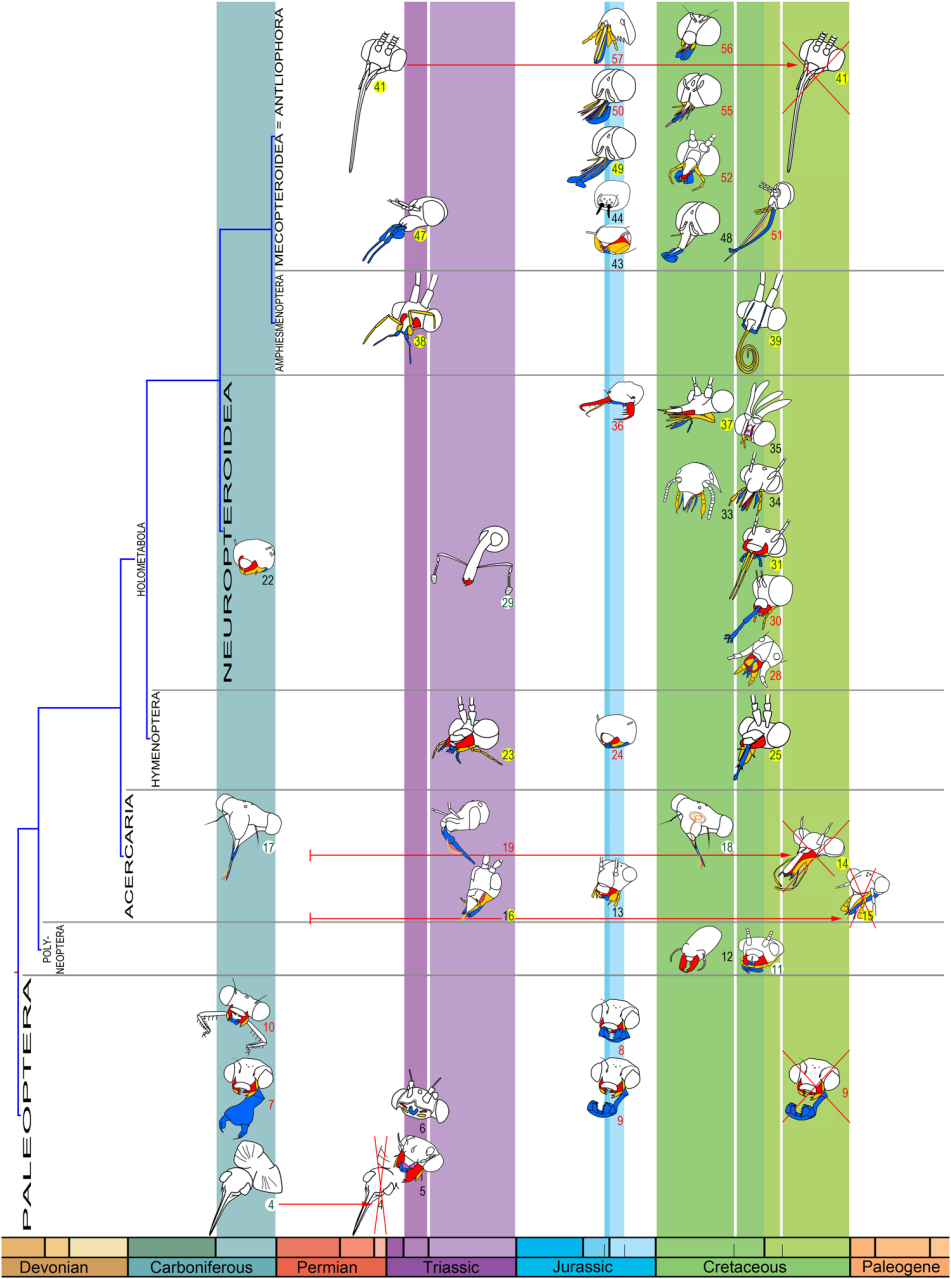
Mouthpart types supporting the six main geological periods of changes in insects. Mouthpart types are schematic (not a species in particular). Colour codes: red: mandibles, orange: maxillae, blue: labium. Numbers correspond to the characters in Matrix Table S1, red: predator types, green: phytophagous types, black undefined or omnivorous types, yellow circles: types feeding on flowers (pollen and/or nectar), white circles: types not feeding on flowers. Mouthpart type 2 is not represented during ‘Late’ Cretaceous as it corresponds to a Hexapoda but not an Insecta. Types 4 and 41 represented for Late Permian and type 15 for Paleocene because they support the hierarchy (discussed in main text). Crossed mouthparts correspond to extinctions. Red arrows: time of duration of extinct mouthpart types. The phylogeny of superordinate taxa is based on^54^. Copyright P.N.

Six geological times are characterized by major diversifications of the types of mouthparts (Figs 2a and 3) i.e., Late Carboniferous, Middle Triassic, Late Triassic, ‘Callovian-Oxfordian’, ‘Early’ Cretaceous, and ‘Albian-Cenomanian’. Other geological times do not present more than two new mouthpart types.

#### Late Carboniferous

The Late Carboniferous is the first time of increase in the diversity of mouthpart types. It is characterized by the first records of feeding apparatus types of Odonatoptera (predatory larvae and adults), endopterygotan larvae type, and basal hemipteran type^25,26^. These types are crucial in extant ecosystems, especially the holometabolous insects which represent today about 85% of the insect species diversity^25^. It is also the time of first record of the fossil palaeodictyopteran beaked mouthpart type.

The Late Carboniferous is indeed known to be characterized by the first winged insects, and the first radiation of the major pterygote clades, especially the Polyneoptera and Acercaria)^7,9,11^. On the contrary fossil Carboniferous Holometabola are rare. For the mouthparts, they appeared in the form of a larva corresponding to the stem-group of Coleoptera^26^, The oldest known ‘true’ beetles are only Early Permian, even if their sister group Skleroptera Kirejtshuk & Nel 2013 is known in the Late Carboniferous^27,28^. Other known Carboniferous Holometabola have their mouthparts not preserved. The rarity of the Carboniferous Holometabola compared to the situation in later periods, suggests that the great differences between their larvae and adults were not a crucial factor of diversification of the clade^9,25,26^.

Some of these mouthparts appear to be linked to phytophagy with the new types from Palaeodictyoptera and Acercaria (Hemiptera), and others to insectivory (Odonadoptera and some Archaeorthoptera^29^. The Late Carboniferous is a period characterized by a great increase in botanical diversity with favorable warm and humid forest environments dominated by ferns, horsetail trees, *Cordaites*, and clubmosses, on a great part of emerged lands. But besides favorable environments for insects, it must be said that there can be a bias for the rise of diversification observed for this epoch. Indeed the Late Carboniferous is rich of deposits with exceptional preservation (viz. Mazon Creek, Commentry, or Avion) (Fig. 2c), and moreover it is flanked by two geological times with less Lagerstätten^9^. Terrestrial arthropods were extremely rare during the Early Carboniferous ‘hexapod gap’^30,31^. The Permian entomofaunas are well known, but several outcrops, especially from the Southern Hemisphere, need further investigations.

#### Middle and Late Triassic

The Middle and the Late Triassic represent the second and third detected periods of diversification of the mouthparts. Most of these are associated with phytophagy, such as the asymmetrical piercing sucking type of Thysanoptera, probably linked to the pollen piercing^32^, and the amphiesmenopteran type (the mandibular lepidopteran type). The curculionid type is putatively adapted to seeds^33^, an innovation promised to a major diversification in plants. The maxillary-labial type of Hymenoptera and the tipuloid type of Diptera, appearing during Middle-Late Triassic, are more precisely linked to nectarivory. The Middle Triassic is also the time of appearance of the segmented beak with high insertion of rostrum of predatory aquatic nepomorphan bugs (Heteroptera).

Hence the first records of the mouthparts of the three nowadays mega-diverse lineages Diptera, Amphiesmenoptera, and Hymenoptera are Triassic. This period is also important for the diversification in Acercaria and Coleoptera, and for the emergence of the crown-Ephemeroptera with nymphs corresponding to modern families.

The emphasis on mouthparts linked to phytophagy during the Middle and the Late Triassic can be correlated with the diversification of the gymnosperms beginning along with the dominance of horsetails, with cycads, gingkos, as well as the onset of flowering plants^34^, especially with Bennettitales and Gnetales owing complex ‘flower’ structures^35,36^ and hard seeds^37^. Here again there can be a bias due to the fact that the Early Triassic and the Early Jurassic that delimitate the Middle and the Late Triassic are relatively poor in insects, contrary to these epochs characterized respectively by the exceptional Lagerstätten of the ‘Grès à Voltzia’ and Madygen.

#### Early Jurassic

Despite the appearances of three mouthpart types, it is not considered as a major period because the two dysticoid and gyrinid larval mouthparts are surely older (Supporting Information).

#### ‘Callovian-Oxfordian’ (Middle-Late Jurassic)

The ‘Callovian-Oxfordian’, during the Middle-Late Jurassic, corresponds to the greatest visible diversification of mouthparts during all geological times (ten new mouthpart types). Most significant are the first records of four new mouthpart types inside Diptera: the larval Orthorrhapha and Muscomorpha, plus adult nemestrinid and tabanid (or rhagionid) types. Moreover one more dipteran mouthpart type appeared in ‘Late’ Jurassic. The siphonapteran type, which we consider in ‘Callovian-Oxfordian’, perhaps dates back to the Late Triassic when the first Pterosauria and Mammals appeared with filaments-like hairs or hairs. It is also the time of the first records of the hymenopteran crown Apocrita larval type, with the onset of new specialized habits inside Hymenoptera such as parasitoid ‘strategy’, and care to larvae leading to (eu-)sociality in bees, wasps, and ants. This is especially linked to the modification of the larval mouthparts because these larvae are unable to actively search their food. It is an important time of diversification for Odonatoptera, with the appearance of two new nymph mouthpart types (short and long spoon-shaped masks) out of the three ever known for the order. It is also the time of the first record of the elongated larval mandibulo-maxillar apparatus of Neuroptera adapted for piercing and sucking. Perhaps the psocopteran mouthpart type dates back to the Permian as the psocopteran-wing type is recorded at that time.

As far as mouthparts are concerned, the Middle-Late Jurassic corresponds to the diversification in preexisting lineages. The Jurassic is cooler than the Early Cretaceous and Triassic, but still warmer than today, suitable for the terrestrial fauna and flora. The diversification of phytophagous insects continued with new insects appearing with specialized mouthparts to eat on the ‘flowers’ of the dominating gymnosperms, especially Bennettitales (*Williamsoniella* Hamshaw, 1915)^38^: the Jurassic nemestrinid and rhagionid flies already had long mouthparts adapted to sucking liquids and were putatively eating on these fossil ‘flowering’ gymnosperms. The descendants of these flies are now pollinating long-tube angiosperm flowers. But we can see that new mouthpart types mainly concerns non-phytophagous insects that experienced diversification surely through diversification of their hosts or preys in the new ecosystems. However the ‘Callovian-Oxfordian’ is also probably the main biased period due to two well-studied deposits with exceptional preservation and very rich faunas, viz. Daohugou and Karatau, while the Early Jurassic, early Middle Jurassic and the Kimmeridgian-Tithonian have not given such very rich outcrops.

#### ‘Early’ Cretaceous and ‘Albian-Cenomanian’

Eight new types ‘appeared’ during the ‘Early’ Cretaceous, viz. the soldier mandibles of eusocial termites, the coleopteran leiodid- and eucinetid-like type, plus four new dipteran types: proboscis of predatory Asilidae, Empidoidea, proboscis of female Ceratopogonidae or Phlebotomidae, and one type deduced by inference from the presence of the muscomorphan pupae. It is also the time of appearance of the lapper neuropteran types of Nemopteridae. The’psyllids’, recorded from this epoch, could be older as they are currently considered as sister group of the (Aphidoidea + Coccoidea)^39,40^, with Permian and Triassic fossils.

The ‘Albian-Cenomanian’ is characterized by the first records of nine new mouthpart types. Five of them concern the Neuropteroidea (Neuropterida + Coleopterida): the Strepsiptera male adult, the coleopteran piercer Cerylonidae, sucker Meloidae (adult), predatory Staphylinidae Steninae, and predatory Lampyridae (larval) types. It is also the time of first records of the phasmatodean type, the lapper-sucker maxillary-labial apoid type, and the Lepidoptera Glossata type. The Culicidae type may be older as mosquitoes mainly fossilize in amber deposits, even if this family is still unknown in the older Lebanese amber.

A great diversification of mouthparts associated with angiosperm flowers occurred from the Early Cretaceous till the Cenomanian, i.e., Neuroptera Nemopteridae, Coleoptera Meloidae, Hymenoptera with maxillar-labial mouthparts, and Lepidoptera with elongate proboscis, all associated with nectarivory. This diversification is even greater than what suggested in our analysis because during the Albian-Cenomanian, there are at least two more new types of mouthparts associated to nectarivory but these are recorded only for this period (Supplementary Information).

Early Cretaceous till Cenomanian is also characterized by the diversification of mouthparts associated with predatory habits, viz. four new beetle mouthpart types other than that of Meloidae, and four new types of fly mouthparts, mainly predatory or parasite, either on other insects (viz. Asilidae, Empidoidea), or on mammals and birds (viz. Ceratopogonidae, Phlebotomidae, Simulidae) (plus a type of ant mouthparts recorded only for the Albian-Cenomanian, see Supporting Information). The Strepsiptera experienced a diversification together with their hymenopteran hosts^41^.

The ‘Early’ Cretaceous has given several very rich Lagerstätten (e.g. Lebanese amber, Crato Formation, Baissa, etc.). The ‘Albian-Cenomanian’ Lagerstätten are the Burmese, Spanish, and French amber. The ‘Late’ Cretaceous is comparatively very poor in fossil insects, especially during Campanian and Maastrichian, due to the lack of outcrops.

### Extinctions

Our study shows that the different types once appeared are generally held to present, few disappearances occurred. This could be explained by: persistence of food sources, strong ‘versatility’ of these morphological types (e.g., ability to obtain pollination drops or nectar on gymnosperms^31^ or on angiosperms), or by ‘plasticity’ (a morphological type undergoing minor adaptations can be used on another food source)^42^.

However the hierarchy points also three breaks with extinctions of mouthpart types (Fig. 2a,b). The extinction of the palaeodictyopteran type appears in the hierarchy at the break between (Late Carboniferous-Early Permian-Middle Permian) and (Late Permian-Early Triassic). Three cases of extinctions of mouthparts characterize the ‘Albian-Cenomanian’, as the types are no longer present during the ‘Late’ Cretaceous, viz. aeschnidiid long spoon-shape masks^43^; Aneuretopsychina (Mecoptera) whipper licker sucker type with long siphon^44^; and permopsocidan mill type, close to the psocodean type but with a great lengthening of labrum and mandibles^45^. The extinction of the lophioneuridan (Thripida) mouthpart type (close to the thysanopteran type, but head highly different) concerns the ‘Late’ Cretaceous (absent in Paleogene).

### ‘Crises’ of biodiversity

Among the major crises that have impacted the global biodiversity on Earth, none seems to have had a significant impact on mouthparts’ fossil record. It is paradoxical that so intense crises be not visible in our results. Indeed, such extinctions are expected to profoundly alter ecosystems, so particularly plant-insect interactions. Possibly still unknown nutritional strategies may have been present before the crises and affected by them, but not preserved in the fossil record. This hypothesis is more plausible for the Permian-Triassic (P-T) crisis (due to the absence of Permian amber and to the incompleteness of the insect fossil record for the Late Permian), than for the Cretaceous–Paleogene (K-T) crisis. Only the extinction of the lophioneuridan type of mouthparts happened during the Latest Cretaceous or at the K-T event. The palaeodictyopteran extinction (and therefore of their mouthpart type), possibly precedes the P-T crisis. It is currently impossible to determine more precisely when it occurred exactly, and if it coincided or not with the P-T. There is no clear record of Palaeodictyoptera in the Latest Permian, and an alleged recently described Triassic Palaeodictyoptera finally resulted into an orthopteran.

The major extinction of insect mouthpart types essentially touches the ‘mid’-Cretaceous, with three mouthpart types lastly seen during the ‘Albian-Cenomanian’. The Odonata were particularly affected by the mid-Cretaceous turn over, with the extinction of the very diverse Aeschnidiidae and their nymph mouthparts (see above). A previous WAPUM analysis based on all odonatopteran morphological structures gave a hierarchical topology affected by a grouping ‘Jurassic’ & ‘Early’ Cretaceous that strongly differs from the more recent epochs^18^ (Fig. S1). This phenomenon could be related to the appearance in the Jurassic of several new odonatan morphological structures that maintained into the ‘Early’ Cretaceous. The transition between the ‘Early’ Cretaceous and the ‘Late’ Cretaceous had a greater impact on the general morphology of Odonata than what we observe on the insect mouthparts in general.

Our data do no put in light any other extinction of mouthpart type. In particular no mouthpart type went extinct during the Late Carboniferous, nevertheless a time associated to a major change in ecosystems leading to the replacement of detritivorous insect communities by taxa feeding on live tissues of the new arborescent plants^11^. It seems that the insects could have adapted the ancient mouthpart types to new resources. Also there is no trace of a Triassic-Jurassic extinction of mouthpart types in our data. It seems that the Triassic-Jurassic crisis did not affect much the insect and plant fossil record at family or order levels^9,46^.

### Comparison between mouthpart disparity and fossil family diversification

The two approaches clearly do not count the same things. There is no strong relation between the morphological disparity (e.g., number of mouthpart types) and the numbers of families (or species) described for a given epoch, because a particular morphological structure can be shared by few species (e.g., the permopsocid mouthparts)^45^, while another can be present in large families or an entire order; also many fossil species and families are based on incomplete fossils for which the mouthpart structures are unknown. It is also possible to have different morphological types in the same family, e.g., the *Stenus* mouthparts differ from those of other staphylinids.

When comparing the main epochs of diversification of mouthparts with those put forth by other taxonomic diversity studies, it appears that the evolution of disparity of the mouthpart types is best congruent with the drawn evolution of net ‘range-through’ family richness (first and last appearances)^15^, with six corresponding periods of major diversifications of mouthparts, viz. Late Carboniferous, Middle and Late Triassic, ‘Callovian-Oxfordian’, ‘Early’ Cretaceous, and ‘Albian-Cenomanian’.

Our results are not congruent with the Permian and the Eocene diversifications^3,4,8,9,15^. We confirm the bias of Eocene diversification due to the ‘over-studied’ Lagerstätten of Baltic, Oise, or Rovno amber, and the palaeolakes of Messel, Green River, Florissant, etc. The bias is also due to the relative lack of information for the latest Cretaceous-Paleocene.

The congruence between the periods of diversification obtained with the two different approaches of disparity and diversity gives some weight to both results.

Our results differ from earlier studies by the relative importance of some periods. We strengthen the Middle-Late Triassic and the ‘Albian-Cenomanian’, as periods of increases of disparity of the mouthpart types, contrary to the studies based on the insect families’ net diversification^3,4,8,10^. In earlier studies, Middle and Late Triassic were not particularly considered as major origination periods when considering the family diversification^7,11^. However the Middle Triassic was also described as the beginning of one the four major herbivore expansions^47^. For the comparison of the ‘Albian-Cenomanian’, we have of course taken into consideration the changes in the estimations of the ages of some important outcrops since these studies, e.g., Karatau was Kimmeridgian but now is Oxfordian. Important changes in taxonomy, improvement in the stratigraphic resolution of family ranges to stages, and extensions of known ranges in fossil families have also occurred since these publications^8^. We also show a diversification of mouthpart types during the Cretaceous Terrestrial Revolution (KTR) ca. 125–85 Ma ago^9^, while the contemporaneous diversification of insect taxa in relation to the angiosperm radiation (Fig. 2d) is a debated matter. Clapham *et al*.^15^ and Condamine *et al*. (therein Fig. S1)^9^ recorded two increases in fossil families’ richness during ‘Early’ Cretaceous and ‘Albian-Cenomanian’, while other studies of family diversification did not recorded any ‘Albian-Cenomanian’ peak of origination^7,11^.

As far as extinctions are concerned, mouthparts study does not allow us to see a profound event of extinction around the P-T boundary (only palaeodictyopteran extinction type, happening after Middle Permian). For most authors the Permian is the landmark of the most profound event in the history of insects, and an event affecting insects not at, but before, the P-T boundary has been previously observed^4,9,11^. It may concern the P-T extinction either *via* the background extinction, *via* the ‘Signor–Lipps effect’^9,11,48^, or via the Capitanian (Middle Permian) extinction event^49^.Whatever the timing of extinction, and as reported for other organisms such as plants, the P-T boundary shows modification of communities: the insect taxon record before and after P-T separates two different faunas at higher ranks^3,11^. Although there is temporal overlap of some lineages, the Late Paleozoic entomofauna consists of ‘basal’ clades of apterygotes, paleopterans, archaeorthopteran, dictyopteroids, and acercarians. The post-Paleozoic insect fauna is mainly characterized by more ‘derived’ clades of Odonata, Orthoptera, Acercaria, and the dominant Holometabola^11^. A first Permian holometabolan radiation occurred, concerning Coleoptera, Neuropterida, and, to a lesser extent, Mecopterida. But these groups were still minority or even absent in some palaeoenvironments (e.g., missing beetles and Mecopterida in the ‘red’ French Middle Permian), which is not the case for the Triassic outcrops in which beetles are always present and diverse.

Our data on mouthpart types are congruent with analyses of the fossil record at family level that show a mid-Cretaceous taxonomic turnover in the insect fauna^3,4,9,11,12^. This can be qualified as an extinction event, concerning many ancient Mesozoic freshwater taxa that were replaced by more modern clades^50^; this could also represent the final extinguishment of ancient mostly phytophagous clades that perhaps were non-competitive in exploiting increased angiosperm food resources^11^.

There is little evidence from the insect fossil record for a significant insect K-T mass extinction^7–9,51^. However the K-T has been proposed to have been responsible for some extinction, e.g., at least North American host-specialist herbivores^11^. Recent analyses of fossil record at family level show a Late Cretaceous negative net diversification^9^. We only register the extinction of the lophioneuridan type of mouthparts.

### Mouthpart disparity vs. taxa diversification based on phylogenetic studies

By using phylogenetic comparative methods (BAMM model) to avoid problems of incomplete fossil record, Condamine *et al*.^9^ did not recorded any significant Cretaceous or Middle-Late Jurassic diversification shifts among taxa, which are the main times of diversification for mouthparts. They recorded only two (out of eight) significant diversification rate shifts which appears congruent with our mouthpart data^9^: one at the Late Carboniferous concerning beetles, and the other for flies during very Early Jurassic. Another significant diversification rate shift is approximately placed during Late Triassic (~−225 Ma), for Hymenoptera. It is considered as being ‘consistent with the development of trophically specialized habits (i.e. parasitoid)’^9^. However our data on mouthpart types show that the mouthpart type of apocritan larva that corresponds surely to parasitoids, has its oldest fossil record only during the ‘Callovian-Oxfordian’. During the Late Triassic only the ‘basal’ hymenopteran family Xyelidae is known. Because the large Hymenoptera fossilize well, Late Triassic seems too early for a diversification of the Apocrita as compared to the fossil record. This time dates rather the appearance of the hymenopteran crown group, with a diversification rather dating from Jurassic. Another study, not supported by the fossil record, placed the diversification of hymenopteran crown group during Permian^52^. Another shift of diversification rate is during the Cenomanian, ~−95 Ma and concerned the Diptera; it is linked to the phytophagous Agromyzidae or Anthomyiidae^9^, at this time we observed numerous appearances of new dipteran mouthpart types linked to predatory habits.

Phylogenetic results showed that the impact of the radiation of the four richest holometabolous orders, Coleoptera, Lepidoptera, Diptera, and Hymenoptera was instrumental in shaping the diversification dynamics of insects^9^. We show that these orders had a high mouthpart diversification during the insects’ evolution (28 types proper to these orders, 49% of the total number of types), congruently with other lineages. Nevertheless the number of types in Coleoptera (nine types) and Diptera (14 types) is clearly more important than for Hymenoptera (three types) and Lepidoptera (two types). The Acercaria (nine types) also greatly diversified their mouthparts compared to Odonatoptera (four types), and Neuroptera (two types).

### Mouthpart types

We renewed the previous analyses on mouthpart types^11,20^. Our study was especially meant to focus on homologous morphological structures. Thereby some differences between Labandeira’s results^11,19^ and our results can be explained by the way that groups were built in both studies^11,19^. For example, Labandeira’s ‘Labellate’ type included some Diptera and the Mecoptera: Nannochoristidae. But nannochoristid’s and tipuloid’s mouthparts are very different (Supporting Information). Similarly Labandeira grouped within the guild ‘Glossate’ the Neuroptera: Nemopteridae and the Hymenoptera: Apoidea and Vespoidea. These two types of licking structures are not homologous. Also the nemopterid type is ‘Early’ Cretaceous while the vespoid-apoid type is ‘Albian-Cenomanian’. Other examples (Tubulomandibulata, Segmented Beak, Hexastyle, etc.) contain non-homologous structures. The evolutionary message carried by new innovations (redundant according to Labandeira, because they would have appeared in previous taxa having the ‘same’ type of mouthparts) is important because it contains information on morphological types appeared or disappeared with taxa bearing them, and consequently information on the selection pressures that affected these taxa. Analyses of the evolution of feeding guilds (particularly in insects) in relation to diversification of angiosperms had shown that by the Middle Jurassic 65 to 88% of all modern insect mouthpart classes were present^4^. Our renewed data set shows that new mouthpart types arose since the ‘Callovian-Oxfordian’ stages (the Lagerstätten Karatau was previously considered as Late Jurassic)^4^, and that important mouthpart types, in particular many linked to nectarivory, arose during the Cretaceous.

Future development of our approach and improvement of the analyses can be made by adding new characters obtained by the study of other organs, i.e., the wings, the legs, and the abdominal terminalia as shown in a similar analysis that was undertaken with the Odonatoptera (Fig. S1). These are also crucial structures for the history of insect evolution.

## Methods

The Maximum Parsimony analysis was performed using the software win-paup4b10, using the options BandB and HSearch. The characters are defined as the ‘presence vs. absence of taxa bearing a type of mouthpart’ (Fig. 1, Table S1). A character coded with ‘1’, corresponds to the presence of a fossil that bears the morphological mouthpart type or that belongs to the crown group bearing it. A character coded with ‘?’ corresponds to the presence of a fossil inside the stem group but with its mouthparts not preserved. The analysis using Wagner Parsimony, with the question marks ‘?’ in the matrix identified 45 most parsimonious hierarchies (Fig. 2a).

### Data availability

All relevant data are available from the authors.

## Electronic supplementary material

Supplementary information
