# Supplementary material for: Diversification of insects since the Devonian: a new approach based on morphological disparity of mouthparts
Source: Sci Rep. 2018 Feb 23;8:3516. doi: 10.1038/s41598-018-21938-1 (PMC5824790; doi:10.1038/s41598-018-21938-1)
Supplement: Supplementary file 1 — Supplementary information [file 41598_2018_21938_MOESM1_ESM.pdf]

Diversification of insects since the Devonian: a new approach based on morphological disparity of mouthparts

Patricia Nel, Sylvain Bertrand & André Nel

SUPPLEMENTARY INFORMATION

Contents.

Table S1. Matrix of stratigraphic levels and characters based on presence vs. absence of taxa bearing a type of mouthpart.

Figure S1. Strict consensus hierarchy obtained after the Wagner parsimony analysis for Odonatoptera (modified after Nel et al.[1]). In the Jurassic and Cretaceous, the short vertical lines correspond to the limits of 'Callovian-Oxfordian' and the 'Albian-Cenomanian'. Copyright Taylor & Francis.

S.1. Material and Methods

Table S1. Matrix of stratigraphic levels and characters based on presence vs. absence of taxa bearing a type of mouthpart. A character coded ‘1’, corresponds to a fossil bearing the morphological mouthpart type or belonging to the crown group bearing it. A character coded ‘?’ corresponds to the presence of a fossil of stem group but with mouthparts not preserved. The ‘outgroup’ OG is a geological epoch containing no character carrier taxon (viz. Silurian or Ordovician).

	1	2	3	4	5	6	7	8	9	10	11	12	13	14	15	16	17	18	19	20	21	22	23	24	25	26	27	28	29	30	31	32	33	34	35	36	37	38	39	40	41	42	43	44	45	46	47	48	49	50	51	52	53	54	55	56	57
Pliocene	1	1	1	0	1	1	1	1	0	1	1	1	1	0	0	1	1	1	1	1	1	1	1	1	1	1	1	1	1	1	1	1	1	1	1	1	1	1	1	1	0	?	1	1	1	1	1	1	1	1	1	1	1	1	1	1	1
Miocene	1	1	1	0	1	1	1	1	0	1	1	1	1	0	0	1	1	1	1	1	1	1	1	1	1	1	1	1	1	1	1	1	1	1	1	1	1	1	1	1	0	?	1	1	1	1	1	1	1	1	1	1	?	1	1	1	1
Oligocene	1	1	1	0	1	1	1	1	0	1	1	1	1	0	0	1	1	1	1	1	1	1	1	1	1	1	1	1	1	1	1	1	1	1	1	1	1	1	1	1	0	?	1	1	1	1	1	1	1	1	1	1	?	1	1	1	1
Eocene	1	1	1	0	1	1	1	1	0	1	1	1	1	0	0	1	1	1	1	1	1	1	1	1	1	1	1	1	1	1	1	1	1	1	1	1	1	1	1	1	0	?	1	1	1	1	1	1	1	1	1	1	0	0	1	1	1
Paleocene	1	1	1	0	1	1	1	1	0	1	1	1	1	0	0	1	1	1	1	1	0	1	1	1	1	1	1	1	1	1	1	0	1	1	1	1	1	1	1	1	0	?	1	1	1	1	1	1	1	1	1	1	0	0	1	1	1
'Late Cretaceous'	1	1	1	0	1	1	1	1	0	1	1	1	1	0	1	1	1	1	1	1	0	1	1	1	1	1	1	1	1	1	1	0	1	1	1	1	1	1	1	1	0	?	1	1	1	1	1	1	1	1	1	1	0	0	1	1	1
Albian-Cenomanien	1	?	1	0	1	1	1	1	1	1	1	1	1	1	1	1	1	1	1	1	0	1	1	1	1	1	1	1	1	1	1	0	1	1	1	1	1	1	1	1	1	?	1	1	1	1	1	1	1	1	1	1	0	0	1	1	1
'Early Cretaceous'	1	?	1	0	1	1	1	1	1	1	0	1	1	1	1	1	1	1	1	1	0	1	1	1	0	1	1	0	1	0	0	0	1	0	?	1	1	1	?	1	1	?	1	1	1	1	1	1	1	1	0	1	0	0	1	1	1
'Late Jurassic'	1	?	1	0	1	1	1	1	1	1	0	0	1	1	1	1	1	?	1	1	0	1	1	1	0	1	1	0	1	0	0	0	?	0	0	1	0	1	0	1	1	?	1	1	1	1	1	?	1	1	0	?	0	0	0	0	1
Callovian-Oxfordian	1	?	1	0	1	1	1	1	1	1	0	0	1	1	1	1	1	?	1	?	0	1	1	1	0	1	1	0	1	0	0	0	0	0	0	1	0	1	0	1	1	?	1	1	0	1	1	?	1	1	0	?	0	0	0	0	1
'Middle Jurassic'	1	?	1	0	1	1	1	0	0	1	0	0	?	1	1	1	1	?	1	?	0	1	1	?	0	1	1	0	1	0	0	0	0	0	0	?	0	1	0	1	1	?	?	0	0	1	1	?	0	0	0	?	0	0	0	0	0
Early Jurassic	1	?	1	0	1	1	1	0	0	1	0	0	?	1	1	1	1	?	1	?	0	1	1	?	0	1	1	0	1	0	0	0	0	0	0	?	0	1	0	1	1	0	?	0	0	1	1	?	0	0	0	?	0	0	0	0	0
Late Triassic	1	?	1	0	1	1	1	0	0	1	0	0	?	1	1	1	1	?	1	?	0	1	1	0	0	?	?	0	1	0	0	0	0	0	0	?	0	1	0	1	1	0	?	0	0	0	1	0	0	0	0	0	0	0	0	0	0
Middle Triassic	1	?	1	0	1	1	1	0	0	1	0	0	?	1	1	0	1	?	0	?	0	1	0	0	0	?	0	0	0	0	0	0	0	0	0	?	0	1	0	1	1	0	0	0	0	0	1	0	0	0	0	0	0	0	0	0	0
Early Triassic	1	?	1	0	?	?	1	0	0	1	0	0	?	1	1	0	1	?	0	?	0	1	0	0	0	?	0	0	0	0	0	0	0	0	0	?	0	0	0	1	1	0	0	0	0	0	0	0	0	0	0	0	0	0	0	0	0
Late Permian	1	?	1	?	?	?	1	0	0	1	0	0	?	1	1	0	1	?	0	0	0	1	0	0	0	?	0	0	0	0	0	0	0	0	0	?	0	0	0	1	1	0	0	0	0	0	0	0	0	0	0	0	0	0	0	0	0
Middle Permian	1	?	1	1	?	?	1	0	0	1	0	0	?	1	1	0	1	?	0	0	0	1	0	0	0	?	0	0	0	0	0	0	0	0	0	?	0	0	0	0	0	0	0	0	0	0	0	0	0	0	0	0	0	0	0	0	0
Early Permian	1	?	1	1	?	?	1	0	0	1	0	0	?	1	1	0	1	0	0	0	0	1	0	0	0	?	0	0	0	0	0	0	0	0	0	?	0	0	0	0	0	0	0	0	0	0	0	0	0	0	0	0	0	0	0	0	0
Late Carboniferous	1	?	1	1	0	?	1	0	0	1	0	0	?	0	?	0	1	0	0	0	0	1	0	0	0	0	0	0	0	0	0	0	0	0	0	0	0	0	0	0	0	0	0	0	0	0	0	0	0	0	0	0	0	0	0	0	0
Early Carboniferous	1	?	1	0	0	0	0	0	0	0	0	0	0	0	0	0	0	0	0	0	0	0	0	0	0	0	0	0	0	0	0	0	0	0	0	0	0	0	0	0	0	0	0	0	0	0	0	0	0	0	0	0	0	0	0	0	0
Late Devonian	1	?	1	0	0	0	0	0	0	0	0	0	0	0	0	0	0	0	0	0	0	0	0	0	0	0	0	0	0	0	0	0	0	0	0	0	0	0	0	0	0	0	0	0	0	0	0	0	0	0	0	0	0	0	0	0	0
Middle Devonian	1	?	1	0	0	0	0	0	0	0	0	0	0	0	0	0	0	0	0	0	0	0	0	0	0	0	0	0	0	0	0	0	0	0	0	0	0	0	0	0	0	0	0	0	0	0	0	0	0	0	0	0	0	0	0	0	0
Early Devonian	1	?	1	0	0	0	0	0	0	0	0	0	0	0	0	0	0	0	0	0	0	0	0	0	0	0	0	0	0	0	0	0	0	0	0	0	0	0	0	0	0	0	0	0	0	0	0	0	0	0	0	0	0	0	0	0	0
OG	0	0	0	0	0	0	0	0	0	0	0	0	0	0	0	0	0	0	0	0	0	0	0	0	0	0	0	0	0	0	0	0	0	0	0	0	0	0	0	0	0	0	0	0	0	0	0	0	0	0	0	0	0	0	0	0	0

Figure S1. Strict consensus hierarchy obtained after the Wagner parsimony analysis for Odonatoptera (modified after Nel et al.[1]). In the Jurassic and Cretaceous, the short vertical lines correspond to the limits of 'Callovian-Oxfordian' and the 'Albian-Cenomanian'. Copyright Taylor & Francis.

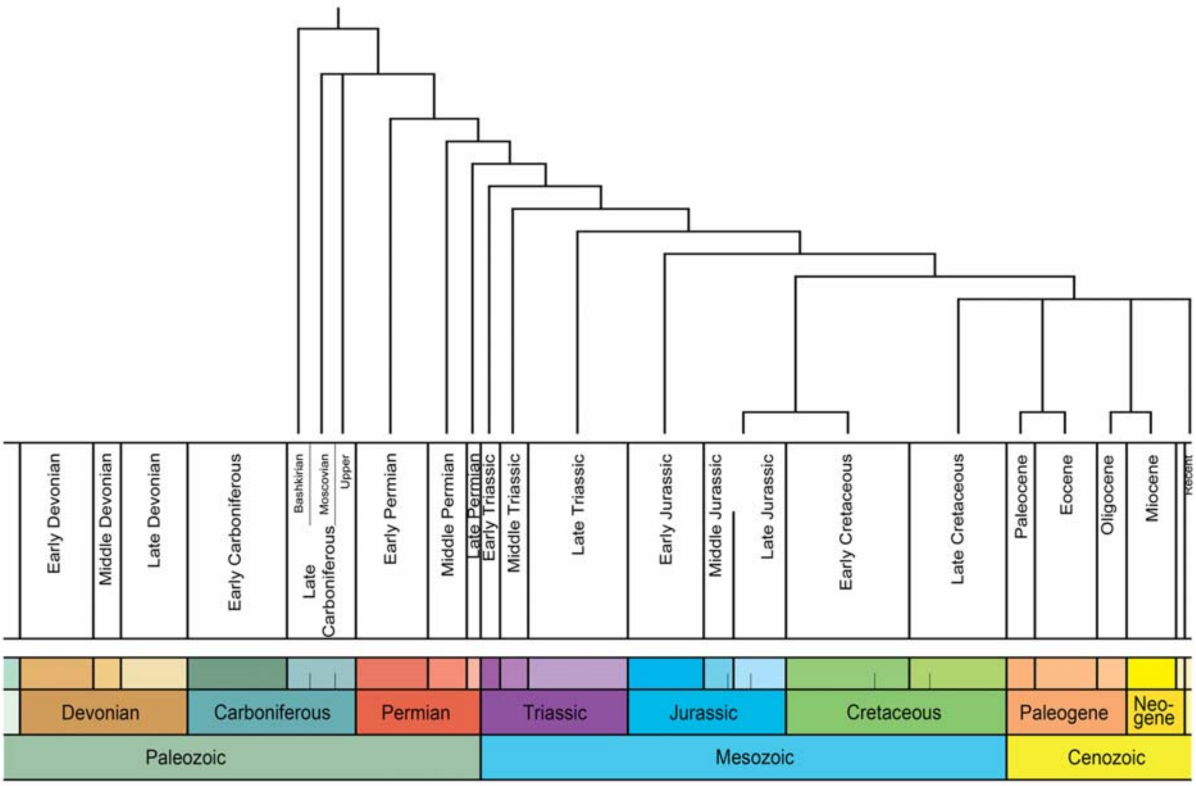

S.1. Material and methods

1.1. Theoretical problems linked with the use of taxonomic diversity

The main problem in analyses based on taxonomic diversity is due to the absence of knowledge on the monophyly of the concerned fossil taxa (families, orders, etc.). Authors[2] argued that monophyly should be considered under a broader sense, i.e. a group containing taxa with a common ancestor, and thus should include paraphyletic groups; and monophyly sensu stricto should be replaced by holophyly. Nevertheless in the particular application to counting the appearances and extinctions of clades, making mixtures of paraphyletic and 'holophyletic' objects results in counting twice (or more), the concerned groups, with resulting potentially significant number of errors. As an example, in a recent 'phylogenetic system' of the Hymenoptera[3], the paraphyletic recent and fossil Megalyridae were supposed to have given rise to the fossil Maimetshidae and Stigmaphronidae plus the paraphyletic modern and fossil Megaspilidae, itself supposed to have given rise to the modern and fossil Ceraphronidae. Under this frame and considering the strict monophyly, these five families should be grouped into a monophyletic 'Megalyridae'. Counting these paraphyletic groups or not implies five appearances plus two extinctions, versus a unique appearance. A more recent phylogenetic analysis resulted in a very different frame[4].

Even if the monophyly of a taxon is well supported, its limits can change with authors, e.g., the rachiberothids are either considered as a monophyletic family or as a berothid subfamily: one family or two?

Many families are monospecific (e.g., Fatjanopteridae, Protoprosbolidae, Ampelipteridae, etc.), while others contain thousands of species with different chorologies, e.g., Chalcididae. The same taxonomic level can correspond to very different biological/ecological information. The extinctions of a monospecific or of a very large family have not the same palaeoecological significance. Lastly such studies are based on the assumption that the (palaeo-)biologies of the

different species in a given family (or even order) are more or less similar (to link extinctions or appearances to environmental changes)[5], which is clearly far from being true.

1.2. Identification of the main types of insect mouthparts

On the basis of a literature review[6-8], we identified 57 'morphological types' of mouthparts, including five now extinct. A 'morphological type' of mouthparts is a set of co-adapted structures, such as the rostrum of a hemipteroid with mandibles and lacinia in stylets included in a tube formed by the labium. The types can be related to the adult stage or to pre-imaginal states. For each of these types, we surveyed, the biological function of the device, the concerned taxa, the correspondence with the morpho-functional types sensu Labandeira[9], and the concerned outcrop(s).

The inventory of the morphological types that we propose is more detailed than that of Labandeira[9], as we tried to approach more accurately the morphological differences. It also contains fossil types unknown at his time. For instance, in Labandeira[9], the type 'segmented beak' concerned all the Hemiptera. For us, these insects are separated into three types (hemipteran 'ground plan', Psylloidea and Aleyrodidae, and Heteroptera) due to the presence of a crumena in Psylloidea and Aleyrodidae and to the high integration of the rostrum in Heteroptera. Thus, among our 57 identified types, only 17 are in bijective correspondence with those of Labandeira[9].

1.3. Hierarchization of the geological epochs using Wagner Parsimony

The 'morphological types' of mouthparts defined as specified above were used to establish a hierarchy of geological epochs from a matrix of characters (Fig 2c). These are defined as follows: 'presence / absence of taxa bearing a particular morphological structure'[1]. The hierarchization is made by Wagner parsimony used in cladistic phylogeny. The 'outgroup'

is a geological epoch containing no character carrier taxon. In this particular case, we chose the Early Devonian, as the first Hexapoda are Middle Devonian. This ‘outgroup’ is used to focus the characters state changes from the absence of the relevant structure to its presence. Reversions (disappearance of a morphological type) are admitted (reversible characters). This allows the hierarchization of the geological periods on the basis of the shared presence of taxa bearing morphological traits.

The chosen objects to hierarchize are the Early, Middle and Late epochs of periods (e.g. Early, Middle and Late Permian), as currently accepted by geologists. However we made two exceptions:

- The Jurassic is currently separated into Early, Middle, and Late Jurassic. But Karatau and Daohugou, the two main hexapod Konservat-Lagerstätten of the late Middle and early Late Jurassic, are dated around the boundary between the two epochs. Therefore we preferred to consider a ‘Middle Jurassic (without Callovian)’ and a ‘Late Jurassic (without Oxfordian)’ and a supplementary ‘epoch’ ‘Callovian-Oxfordian’.
- The Cretaceous is usually separated into Early and Late Cretaceous. We divided the period in ‘Early Cretaceous (without Albian)’, ‘mid-Cretaceous’ grouping the Albian with the Cenomanian and ‘Late Cretaceous (without Cenomanian)’. The ‘Albian-Cenomanian’ seems to be a key period of transition and renewal for the flora and entomofauna[9]. The spelling ‘mid-Cretaceous’ is not in relation with the position of the ‘Albian-Cenomanian’, clearly not in the middle of the Cretaceous in term of duration, but because this period is between the Early and the Late Cretaceous.

The chosen epochs have often very different durations, which may introduce biases in the analysis, but on the other hand, they correspond to relatively stable planetary ecosystem units, separated by major changes in flora and fauna. We did not consider shorter geological

subdivisions (stages) because the fossil record of insects is not complete or accurate enough to be equally recorded for all geological stages.

The Wagner Parsimony analysis was performed using the win-paup4b10 software with the options 'branch and bound' and Heuristic Search, obtaining the same results. The matrix entry software is Nexus. All characters are reversible and of equal weight. The equally most parsimonious hierarchies are equivalent, and are translated through a strict consensus hierarchy.

The present analysis differs from those proposed by Nel et al.[1,10] by the use of characters of the type 'presence of a taxon bearing a complex morphological structure involving multiple organs', and not of characters of the type 'presence of a taxon bearing a single structure modified in a particular way' (e.g., 'stylate lacinia'). This choice is based on the assumption that the insect mouthparts consist of highly interdependent structures. This is generally the case; although there are taxa for which the mouthparts only differ by one or two structures (the Psocodea and Permopsocida differ mainly in the clypeus and lacinia, but implying different feeding habits[11]).

Around 65-67% of the modern hexapod families are known in the fossil record[12-13], thus modern morphological data can be often inferred to the fossil record. We informed the presence of a morphological type for a particular epoch by two very different methods, sometimes complementary, i.e., by direct observation on the fossil of the morphological type in question, or by inference by phylogenetic analysis. The collated data in the matrix are therefore of several origins: direct examinations and/or observations in the literature of fossils bearing particular structures, or else phylogenetic inference drawn from the existence of a fossil taxon belonging to the crown group of a clade in which all (or almost all, except in cases of particular changes in modern subclade) taxa are bearing the same type of mouthparts. These two ways for capturing the data match those defined by Bryant & Russell[14] and Nel[15]. It is also possible to make a phylogenetic inference of the presence of a structure in a fossil belonging to the stem

group of a clade, but the result will be significantly more doubtful. The presence of a morphological type in the oldest fossil of the stem group of a clade is coded with '?' when its mouthparts are not preserved (dubious but possible) in the matrix, while its presence in the oldest fossil of the crown group (with all taxa having the same mouthpart type) is coded with a '1'. The use of the Wagner parsimony method allows the inclusions of '?' in the matrix, which is not possible using phenetic methods.

In the case of an extinct type of mouthparts, we endeavored to find the most recent and the oldest representatives of the taxonomic group actually bearing this type of mouthparts. For example, although Palaeodictyopteroidea are probably present in the early Late Permian, the most recent representative of this group with preserved mouthparts is Middle Permian. The type 'Palaeodictyoptera' is encoded with a '?' for the Late Permian, because it is not absolutely sure that all the Palaeodictyopteroidea had the same mouthparts.

The incompleteness of the fossil data remains an important bias for all these analyses, based on morphological disparity or taxonomic diversity. For instance, Makarkin[16,17] described a sisyrid in a new subfamily Paradoxosisyrinae and a Dilaridae, both from the mid-Cretaceous Burmese amber and both with highly specialized long siphonate mouthparts. Also Barden & Grimaldi[18] cited a new type of mouthparts for Albian-Cenomanian ants. They correspond obviously to unique types of mouthparts, but we currently ignore when they appeared and when they became extinct, more precisely if it was present during the 'Early Cretaceous'. Therefore we cannot use this new information in our analysis. The unique way to partially solve such problems is to find and describe more fossils.

1.4. Characteristic of the obtained most parsimonious hierarchies

The matrix of characters was made using Nexus (Table S1). All characters are equally weighted and unordered. The Wagner parsimony analysis was performed using the software

winPaup4b10, bandb search option. The analysis using Wagner Parsimony, with question marks '?' in the matrix identified 45 most parsimonious hierarchies with the following characteristics: tree length = 57 steps; consistency index = 0.912; retention index = 0.986, rescaled consistency index = 0.899.

1.5. Descriptions of the types of mouthparts (characters) used in the matrix

In some cases, we have grouped different families for the same mouthpart type because these families are closely related and show very few differences between them. In general, only the external structures are considered, but in some cases, internal structures are also considered by phylogenetic inferences, although they are not visible in fossil material.

1. Entognathous type (Collembola, adults): Middle Devonian[9]. ['entognathatous' type of Labandeira & Eble[12]]. (Entognathous structures visible in the fossils of *Rhyniella*).

2. Type 'Entognathous-stylate' of Labandeira & Eble[12]: present in the Collembola Neanuridae known in the Late Cretaceous but most likely very old, probably Devonian after the phylogenic position.

3. Ectognathous type with dicondylic mandibles (Archaeognatha, Zygentoma, Pterygota, adults): Early Devonian[9]. ['adult-ectognathate' type of Labandeira & Eble[12]]. These authors considered a monocondylate type alleged to be present in Archaeognatha, known since the Middle Devonian, but Blanke et al.[19] discovered that the Archaeognatha are in fact dicondylic. The oldest alleged dicondylic hexapod, *Rhyniognatha hirsti* was recently put in doubt as an insect[20].

4. Palaeodictyoptera (adults) (mouthparts shaped as a rostrum of suckers and lickers)[21]: Palaeodictyoptera are known from the Late Carboniferous to Middle Permian, however fossils with preserved mouthparts are known only from the Late Carboniferous to Middle Permian. Other fossils are isolated wings[22-23].

5. Ephemeroptera (nymphs) (hypopharynx with two surnumary lobes): ephemeropteran nymphs of modern type are known in the Middle Triassic (Anisian, Vosges), other taxa belonging to the ephemeropteran stem group are known in the Permian, but nothing is known concerning their mouthparts. [‘pectinate’ type of Labandeira & Eble[12]]

6. Ephemeroptera (adults and subimago) (reduced and buccal pieces forming a ‘buccal field’): presence of the modern family Siphlonuridae in the middle Triassic (Vosges)[24]. Nothing is known about the mouthparts of the Palaeozoic ephemeropteran stem group [‘non-trophic’ type of Labandeira & Eble[12]].

7-9. Odonata (nymphs): Three types of masks. The flat masks are presents since the Late Carboniferous[25]; the short spoon-shape masks are presents in the Callovian-Oxfordian, by phylogenetic inference[26]; the long spoon-shape masks, typical of the Aeschnidiidae, are known between the Callovian-Oxfordian and the beginning of the Late Cretaceous[27].

10. Odonata (adults) [‘raptorial-ectognathate’ type of Labandeira & Eble[12]]: carnivorous mouthparts of mill type, with legs modified into a ‘prey capture trap’. Type known since the Late Carboniferous.

11. Phasmatodea (mill type with split labrum): present in a fossil stick insect from the Burmese Albian-Cenomanian 'mid' Cretaceous amber (pers. obs.).

12. Isoptera: Kalotermitidae (soldiers): the type goes into the Early Cretaceous[28]. The mandibles are strongly developed. This type of mandibles, present in all extant kalotermitid soldiers, can be deduced from the presence of the family in the Early Cretaceous.

13. Psocodea 'Psocoptera' (adultes) (lacinia of maxilla is an elongate, strongly sclerotized rod, proximally sunken well into head capsule, apically variously toothed): this type is present in the Oxfordian Jurassic (Karatau, Kazakhstan)[29], viz. the Paramesopsocidae, a Mesozoic family belonging to the clade Psocomorpha, thus with the mouthpart structures of the Psocoptera, by phylogenetic inference for the Jurassic taxon and directly for an Early Cretaceous species from Lebanon. During Permian, *Parapsocidium uralicum* (Psocidiidae) is potentially attributable to the Permopsocida (on the basis of the wing venation), but its mouthparts are poorly preserved[30]. The Permopsocida are not Psocodea, but another acercarian order, with different mouthpart morphology (coded below). The Permian *Zygopsocus permianus*, and the Late Carboniferous *Westphalopsocus pumilio*, both belonging to the psocodean stem group[31-32], are only known by wings. The Permian Hypoperlida, sister group of the Acercaria have mouthparts looking like those of the Psocodea, but the structure of their lacinia is unknown. It is not possible to make a phylogenetic inference for their mouthpart morphology. ['mortar-and-pestle' type of Labandeira & Eble[12]].

14. Acercaria: Permopsocida (adults): this type is close to the Psocodea 'Psocoptera' type. It is characterized by the lengthening of the labrum and mandibles and the middle part of clypeus membranous. They were swallowing pollen and maybe nectar sucking, despite their mandibles

with strong teeth, similarly to the modern nectar feeding *Glomeremus orchidophilus* (Orthoptera: Gryllacrididae)[33]. It is known since the Early Permian (*Dichentomum tinctum*), to the 'mid-Cretaceous'[11].

15. Lophioneurida (adults) (only the left mandible functional and modified distally to a stylet; stylet-like lacinia, mouthparts not orthognathous): by phylogenetic inference, we can estimate that this type is present in the Permian Thripida, but uncertain in the Thripida recorded in the Late Carboniferous[34].

16. Mouthparts of the type 'modern Thysanoptera' (only the left mandible functional and modified distally to a stylet; stylet-like lacinia, mouthparts orthognathous) were described from the Middle Jurassic[35-36] but the clade is present since the Late Triassic. [The type 'Thysanoptera' corresponds to the 'mouthcone' type of Labandeira & Eble[12]].

17. Hemiptera, general type (adults) (piercing-sucking mouthparts with labial segmented rostrum guiding two pairs of respective mandibular and maxillar stylets): Nel et al.[31] cited a wing of Euhemiptera, belonging to the crown group, in the Late Carboniferous; a phylogenetic inference can be done on its mouthparts. Bekker-Migdisova[36] described hemipteran mouthparts in the Permian. [type 'segmented beak' of Labandeira & Eble[12], who did not separate it from the heteropteran and psyllid types].

18. Hemiptera: Psylloidea, Coccidae, and Aleyrodidae (when not used stylets are coiled within an integumental fold, the crumena): The Protopsyllididae are known in the Early Cretaceous (Lebanese amber, Azar, comm. pers.). Aleyrodinae are described from the Early Cretaceous[37]. Taxa of the stem group are known from the Middle Permian, but without precise information

on the mouthparts. The homology of the crumena of Psylloidea, Aleyrodidae and Coccidae is not clearly established, but as these taxa are closely related, we prefer to consider only one character, instead of three.

19. Hemiptera Heteroptera: this type is characterized by a high insertion of the rostrum, more distant from the anterior coxae than in the other Hemiptera. The oldest known Heteroptera are aquatic Nepomorpha (cf. Belostomatidae) from the Upper Triassic of Virginia, with well-preserved mouthparts[38]. The clade is certainly older (Triassic or Permian?) because the Nepomorpha are not the most 'basal' sub-clade of the Heteroptera (the Enicocephalomorpha are the best candidate for this position, but these are very tiny insects only known by fossils in Lebanese Cretaceous amber; the oldest insectiferous amber is Triassic but since now without Hemiptera).

20. Hemiptera: Coccoidea (male adults) (buccal pieces absent, perforated but nonfunctional mouth): Gullan & Cook[39] and Vea and Grimaldi[40] indicated that they were already diversified in the Early Cretaceous. Shcherbakov[41] cited two Late Jurassic adult males, undescribed, after Koteja (pers. comm.). Shcherbakov[41] also cited fossil Naibiidae in the Triassic, Jurassic and Paleocene, but the fact that the Naibiidae are true Coccoidea is debatable[42]. Vea and Grimaldi[40] dated the coccomorphan stem group in the Triassic, after a total-evidence phylogenetic analysis. Triassic fossils are isolated wings or incomplete bodies.

21. Phthiraptera (adults): the unique accurate fossil is Middle Eocene (Messel), with relatively well-preserved head and mouthparts[43]. [type 'buccal cone' of Labandeira & Eble[12]].

22. Holometabola larva 'with reduction of mill type' (larvae) (fusion with labial-hypopharyngial complex): known in the Late Carboniferous[32,44]. [type 'larval-ectognathatous' of Labandeira & Eble[12]]. The presence of the internal labio-hypopharyngial fusion cannot be ascertained on fossil larvae, but as it is present in all the larvae of the holometabolan crown-group, we can infer it in the fossil record.

23. Hymenoptera (adults): the 'ground plan' of the hymenopteran mouthparts is certainly present since the Upper Triassic, supported by the presence of Xyelidae at that time[45]. [type 'maxillolabiate' of Labandeira & Eble[12]].

24. Hymenoptera Apocrita (larvae) (maxilla and labium are simple, soft, unarticulated lobes): this clade is recorded by adults of extinct Ephialtitidae (stem group) from the Lower Jurassic of Germany[46]. The character can be inferred by the presence of representatives of the crown group in the Callovian of China[47].

25. Hymenoptera (adults): lapper-sucker type with maxillo-labial proboscis present in Late Cretaceous Apidae and Vespidae, in fact known in the Burmese amber (Late Albian)[48]. [type 'glossate' of Labandeira & Eble[12]].

26. Coleoptera: Dytiscoidea (larvae) (mandibulate sucker with cibarial and pharyngeal pump): the larva of *Angaragabus jurassicus* (Lower Jurassic) corresponds to a Dytiscoidea with dytiscid mouthparts[49]. Georges[50] confirmed the presence of larvae of this type in the Early Cretaceous. The Dysticoidea are known since the Upper and Lower Triassic (in particular the larva of *Colymbothetis antecessor* and *Protodytiscus johillaensis* but their mouthparts are poorly known)[51-52]. The adults are recorded in the Permian[53].

27. Coleoptera: Gyrinidae (larvae) (mandibulate sucker with cibarial pump): adults of the stem group are recorded from the Upper Triassic, but the crown group is known from the Lower Jurassic[53-54]. Labandeira & Eble[12] put together the 'gyrinid' and the 'dytiscid' types under the name 'tubulomandibulate'. The cibarial pump is not visible in the fossils, so we can only make an inference after the phylogeny.

28. Coleoptera: Lampyridae (larvae) (mandibulate sucker with closed mandibular canal): adult Lampyridae: Luciolinae are known in the Eocene (Baltic amber) and the 'mid-Cretaceous Burmese amber'[55-56], a phylogenetic inference can be done for the larval mouthparts because Luciolinae are belonging to the crown group.

29. Coleoptera: Curculionidae (adults) (mill type at the extremity of elongated cephalic capsule): Gratshev & Zherikhin[57] indicated that the Upper Triassic Obrieniidae have 'curculionoid' mouthparts. [type 'rhyncophorate' of Labandeira & Eble[12]].

30. Coleoptera: Staphylinidae genus *Stenus* (adults) (exsertile elongated labium): the Late Cretaceous species attributed to *Stenus* have their mouthparts not preserved[58]. Zhang & Zhou[59] dated it into the Late Cretaceous through a molecular phylogenetic analysis. Zyla et al.[60] dated this mouthpart type was found in the 'mid-Cretaceous' Burmese amber.

31. Coleoptera: Meloidae and Ripiphoridae (adults) (sucker, elongated maxillary palps): Engel[61] indicated the presence of Meloidae (triungulin larvae) in the Baltic amber Eocene, corresponding to the modern clade, having certainly mouthparts of modern type. Poinar & Brown[62] described a triungulin larva from the Burmese amber. Representatives of the crown

group of the Ripiphoridae are known in the Albian – Cenomanian[63-64]. [The terrestrial Meloidae and Ripiphoridae are of the 'siphonomandibulate' type of Labandeira & Eble[12]]. Meloidae and Ripiphoridae are considered as sister groups, but this hypothesis was recently questioned[65].

32. Coleoptera: Meloidae nectar-feeding type Nemognathini[66] (adult) (sucker, elongated maxillary galea): Known in the Eocene[61].

33. Coleoptera: Eucinetidae and Leiodidae (adults) (piercing lacinial and hypopharyngial stylets, guided by the labrum): the Upper Jurassic Mesocinetidae is a family close to the Eucinetidae[67,68]. Other Early Cretaceous Eucinetidae are currently considered as Coleoptera incertae sedis[69]. Leiodidae without preserved mouthparts are described in the Late Jurassic and the Early Cretaceous[70]. The inference is made after the phylogenetic affinities of the fossils to Leiodidae. [type 'ectognathous stylate' of Labandeira & Eble[12], who added also the Orthoptera: Cooloolidae in it, but this family is unknown in the fossil record].

34. Coleoptera: Cerylonidae (adults) (elongated piercing mandibles, maxillary lacinia and galea, labium, and labrum): Burmese amber (A. Kirejtshuk, pers. comm.).

35. Strepsiptera (modern type of adults): they are described in the 'mid-Cretaceous' Burmese amber[71-73]. Their mouthparts are not typical of the modern Strepsiptera, because they have retained mill type mandibles.

36. Neuroptera (larvae) (sucker mandibulo-maxillate): the oldest Neuroptera are dated from the Early Permian (Prokop, pers. comm.), but these are isolated wings, belonging to the stem group. The oldest representatives of the modern families (crown group) are from the Chinese

Callovian. Thus a phylogenetic inference can be made for this period. For earlier epochs, we have only doubtful inferences, after the presence of wings of representative of extinct families. [‘fossate complex’ type of Labandeira & Eble[12]].

37. Neuroptera: Nemopteridae (adults) (The brush-shaped laciniae, galeae and maxillary palpi form a functional unit that can be extended by the action of the cardo-stipes joint[74]): Labandeira & Eble[12] considered the ‘glossate’ type to encompass adult Neuroptera Nemopteridae. These insects feed on the angiosperm flowers but their mouthpart morphology strongly differs from those of the Apoidea (among other characters, by the proportions of galea / lacinia)[74-75]. The Nemopteridae are recorded in the Early Cretaceous (Crato Formation, Brésil)[76], but their mouthparts are poorly preserved, so we can only make a phylogenetic inference. Aquatic angiosperm flowers are known from this outcrop.

38. Trichoptera + Lepidoptera ‘with mandibles’ (adults): it corresponds to the ‘ground plan’ of the (Trichoptera + Lepidoptera), clade present in the Middle Triassic[77]. This type corresponds to that of the Lepidoptera ‘with mandibles’, known in the Lower Jurassic[78-79]. There is a problem of discrimination between the Trichoptera and Lepidoptera in the Jurassic, Triassic, and even Permian[80]. Many fossils of these epochs can be only attributed to the stem group (Trichoptera + Lepidoptera). Gao et al.[81] described a Rhyacophilidae, while Wang et al.[82] described a Philopotamidae from the Middle Jurassic of China. So, phylogenetic inference of the presence of mouthparts of the modern type can be made for that epoch. Wings of Philopotamidae were described in the Lower to Middle Jurassic[83-84]. Mouro et al.[85] indicated the presence of putative caddisfly cases in a Permian marine environment. The attribution of these ichnofossils to insects is very dubious. [‘haustoriate’ type of Labandeira & Eble[12]].

39. 'True' Lepidoptera (adults): Huang Di-ying (pers. comm.) indicated the presence of an undescribed Lepidoptera with a proboscis in the Burmese amber. The presence of Lepidoptera Glossata with genuine proboscis is uncertain for the Jurassic, despite a record by Kozlov[86] for which Kristensen & Skalski[87] indicated it should be verified. Grimaldi & Engel[8] figured a caterpillar from the Early Cretaceous Lebanese amber that they attributed to the Glossata. The presence of a proboscis in the corresponding unknown adult is not accurate. Labandeira[88] listed Glossata in the Early Cretaceous Crato Formation and from Baissa, but only Eolepidopterigidae and Micropterigidae are described from these outcrops[89]. [type 'siphonate' of Labandeira & Eble[12], even if a mistake in the text of these authors indicated 'siphonate' for the Meloidae].

40. Mecoptera (adults): Riek[90] figured elongated mouthparts for the Late Permian *Choristotanyderus* (Australia). [type 'rostrate' of Labandeira & Eble[12]].

41. Mecoptera with long siphon, type whipper licker sucker (adults) (bifid labrum, reduced mandibles, and elongated maxillae and labium): it is a fossil type present in the clade Aneuretopsychina[91], between the Permian-Triassic (Nedubroviidae)[92], and the 'mid-Cretaceous' (Burmese amber).

42. Mecoptera: Nannochoristidae: a 'labellate' type that differs from those of the Diptera. This family is known in the Middle Jurassic but by extinct genera with poorly known mouthparts[93], thus the inference from the recent to the fossil record is uncertain.

43. Diptera (larvae of hemicephalous type, typical of the Brachycera Orthorrhapha): this type of larva is known for a Callovian Athericidae (China) (Jun Chen, pers. comm.). Adult Athericidae are known in the Late Jurassic of Australia[94]. The Tipuloidea have also this type of

larva; representatives of the tipuloid crown group are known from the Lower Jurassic[95]. Shcherbakov et al.[96] described Late Triassic adult representatives of the stem group of Tipuloidea. [type 'mouthhook' of Labandeira & Eble[12]].

44. Diptera (larvae of acephalous type): Muscomorpha type, recorded by an Oxfordian Jurassic adult Acroceridae from Karatau[97], so we can make a phylogenetic inference. [type 'reduced trophic' of Labandeira & Eble[12], for the Acroceridae].

45. The acephalous larvae of Diptera of the 'Cecidomyiidae' type are known in the Late Jurassic[98]. [type 'mouthhook' of Labandeira & Eble[12]].

46. Diptera: aquatic filter crusher larval types (Culicidae, Simuliidae, Chironomidae, Ceratopogonidae, and Phlebotomidae). Oldest representatives of the Ceratopogonidae are Early Jurassic. This type of mouthparts is present in these different culicomorphan families, more or less related[99]. We provisionally consider them as belonging to only one type.

47. Diptera: Tipuloidea (adults): the oldest records of mouthparts are Lower Jurassic; for older epochs (Middle Triassic), this clade is known by isolated wings[100]. Labandeira & Eble[12] put together this type of mouthparts with those of the Brachycera of muscid type and Mecoptera: Nannochoristidae, in the 'labellate' type. We have separated the muscid type from the tipulid type (partial fusion of stipes). The Nannochoristidae have mouthparts strongly different from those of the Tipuloidea [compare Schneeberg & Beutel[101] with Beutel & Baum[102]].

48. Diptera (adults), adult mouthparts of the *Calliphora* and *Musca* type: McAlpine[103] cited the presence of pupae of calliphorid type in the Late Cretaceous, but no adult. This type is also

present in the Syrphidae, Drosophilidae, etc., some of these clades are known in the Early Cretaceous[8].

49. Diptera (adults), adult mouthparts with elongate proboscis: the Nemestrinidae are the oldest known representatives of this type, their mouthparts are known in the Callovian Jurassic of China and the Oxfordian Jurassic of Karatau[104-105]. This type is also known in the Bombylioidea, a closely related clade. They are nectarivorous. The description of this type is done by Karolyi et al.[106] for the Nemestrinidae and by Szucsich & Krenn[107] for the Bombyliidae.

50. Diptera: Tabanidae (adults) (piercing elongated mandibles, maxillary lacinia, and hypopharynx): Mostovski et al.[108] indicated some Early Cretaceous Tabanidae, with preserved mouthparts. They correspond to a part of the 'hexastylate' type of Labandeira & Eble[12]. These last authors separated this type from derived types in Tabanomorpha in which mandibles and/or maxilla are absent (such as in some Rhagionidae, the Anisopodidae, Mydidae, Therevidae, and Ironomyiidae), but we did not here because they appear to be derived structure inside the clade. Zhang[109] described Callovian Rhagionidae from China.

51. Diptera: Culicidae (adults) (elongated rostrum guiding stylet like mandibles, maxillary lacinia, and hypopharynx): the biting mouthparts of *Burmaculex antiquus* (Burmese amber) corresponds to that of a biting female[110]. It corresponds to the 'hexastylate' type of Labandeira & Eble[12].

52. Diptera: (adults Ceratopogonidae and Phlebotomidae) (scissor like mandibles, cutting labrum): these clades are recorded in the Early Cretaceous[111] by well-preserved fossils with

mouthparts in amber. Simuliidae (adults): Currie & Grimaldi[112] described them from the Turonian of the New Jersey. The pupae are known in the Early Jurassic[113]. [All correspond to the 'hexastylate' type of Labandeira & Eble[12]].

53. Diptera: Muscidae of the type *Stomoxys* (adults) (no stylet like mandibles or maxilla, but labellum teeth): no fossil record. The clade should be dated from the Oligocene on the basis of a molecular dating[114].

54. Diptera: Hippoboscoidae (Hippoboscidae, Streblidae, Nycteribiidae) (adults) (mouthparts equipped with a series of elaborate cheliceral blades at the apex of the labial thecum[115]: the Streblidae are dated from the Oligocene-Miocene[117], the Hippoboscidae are Oligocene[118]. So phylogenetic inferences can be made. The Nycteribiidae seem to be unknown in the fossil record. This type is equivalent to the 'tubulostylate' sensu Labandeira & Eble[12]. These authors added the Glossinidae in this type, also Oligocene[119].

55. Diptera: Asilidae (adults) (without mandibles, with perforating hypopharynx): this family is cited (with modern mouthparts) from the Turonian amber of the New Jersey and from the Early Cretaceous Crato Formation[118-119]. Labandeira & Eble[12] included them among 'monostylates/distylates' together with the Bombyliidae, Acroceridae, and Empididae. As they are carnivorous, we preferred to separate them from the Bombyliidae and Acroceridae, nectarivorous (see above).

56. Diptera: Empidoidea (adults) (without mandibles, the distal part of epipharynx extends laterally with short and cutting plaques, called *epipharyngeal blades* or *epipharyngeal*

armatures, which represent the piercing organs). The Empidoidea are also carnivorous, recorded from the Early Cretaceous[120] and with some doubt, from the Upper Jurassic.

57. Siphonaptera (adults): Siphonaptera of the stem group are present in the Callovian Jurassic of China[121], their mouthparts are of the flea type.

References

1. Nel, A. et al. The Wagner Parsimony using Morphological Characters: a new method for palaeosynecological studies. *Annales de la Société Entomologique de France* (N.S.) **46**, 276–292 (2010).
2. Hörandl, E. & Stuessy, T. F. Paraphyletic groups as natural units of biological classification. *Taxon* **59,** 1641–1653 (2010).
3. Rasnitsyn, A. P. Superorder Vespidea Laicharting, 1781. Order Hymenoptera Linné, 1758 (= Vespida Laicharting, 1781). pp. 242–254. *In*: Rasnitsyn, A. P. & Quicke, D. L. J. (eds). *History of insects*. Kluwer Academic Publishers, Dordrecht, Boston, London, xi + 517 pp. (2002).
4. Vilhelmsen, L. et al. Past and present diversity and distribution in the parasitic wasp family Megalyridae (Insecta: Hymenoptera). *Systematic Entomology* **35**, 658–677 (2010).
5. Condamine, F. L. et al. Global patterns of insect diversification: towards a reconciliation of fossil and molecular evidence? *Nature Scientific Report* **6**, 19208 (2016).
6. Bitsch, J. Système squeletto-musculaire de la tête des insectes. *In*: Grassé, P.-P. (ed.). Traité de Zoologie, Anatomie, systématique, biologie, Insectes. Tête, Aile **8**, 3–41 (1973).

7. Chaudonneret, J. Les pièces buccales des insectes. Thème et variations. *Edition hors-série du Bulletin Scientifique de Bourgogne*, 1–256 (1992).

8. Grimaldi, D. A. & Engel, M. S. *Evolution of the insects*. Cambridge University Press, xv + 755 pp (2005).

9. Labandeira, C. C. Insect mouthparts: ascertaining the paleobiology of insect feeding strategies. *Annual Review of Ecology and Systematics* **28**, 153–193 (1997).

10. Nel, A. et al. Evolution and palaeosynecology of the Mesozoic earwigs (Insecta: Dermaptera). *Cretaceous Research* **33**, 189–195 (2012).

11. Huang, Di-Ying et al. New fossil insect order Permopsocida elucidates major radiation and evolution of suction feeding in hemimetabolous insects (Hexapoda: Acercaria). *Nature Scientific Reports* **6**, 23004 (2016).

12. Labandeira, C. C. & Eble, G. J. The fossil record of insect diversity and disparity. pp. 1–54. *In*: Anderson, J. et al. (eds.). *Gondwana alive: biodiversity and the evolving biosphere*. *Santa Fe Institute Working Paper* **121** (**2000)**.

13. Nicholson D. B. et al. Changes to the fossil record of insects through fifteen years of discovery. *PLoS One* **10** (7), e0128554 (2015).

14. Bryant, H. N. & Russell, A. P. The role of phylogenetic analysis in the inference of unpreserved attributes of extinct taxa. *Philosophical Transactions of the Royal Society of London* **337**, 405–418 (1992).

15. Nel, A. The probabilistic inference of unknown data in phylogenetic analysis. pp. 305–327. *In*: Grandcolas, P. (ed.). The origin of biodiversity in insects: phylogenetic tests of evolutionary scenarios. *Mémoires du Muséum National d'Histoire Naturelle* Paris **173** (1997).

16. Makarkin, V. N. Enormously long, siphonate mouthparts of a new, oldest known spongillafly (Neuroptera, Sisyridae) from Burmese amber imply nectarivory or hematophagy. *Cretaceous Research* **65**, 126–137 (2016).
17. Makarkin, V.N. New taxa of unusual Dilaridae (Neuroptera) with siphonate mouthparts from the mid-Cretaceous Burmese amber. *Cretaceous Research* **74**, 11–22 (2017).
18. Barden, P. & Grimaldi, D. Adaptive radiation in socially advanced stem-group ants from the Cretaceous. *Current Biology* **26**, 1–7 (2016).
19. Blanke, A. et al. Mandibles with two joints evolved much earlier in the history of insects: dicondyly is a synapomorphy of bristletails, silverfish and winged insects. *Systematic Entomology* **40**, 357–364 (2015).
20. Haug, C. and Haug, J.T. The presumed oldest flying insect: more likely a myriapod? *PeerJ* **5** (e3402), 1–16 (2017).
21. Laurentiaux, D. Présence d'un rostre eugereonien chez le paléodictyoptère *Stenodictya lobata* Brongniart. Affinites des protohémiptères. *C. R. Acad. Sci. Paris* **234**, 1997–1999 (1952).
22. Li, Yong-jun et al. A new palaeodictyopterid (Insecta: Palaeodictyoptera: Spilapteridae) from the Upper Carboniferous of China supports a close relationship between insect faunas of Quilianshian (northern China) and Laurussia. *Alcheringa: An Australasian Journal of Palaeontology* **37**, 487–495 (2013).
23. Prokop, J. et al. A new genus and species of Breyeriidae and wings of immature stages from the Upper Carboniferous, Nord-Pas-de-Calais, France (Insecta: Palaeodictyoptera). *Insect Systematics & Evolution* **44**, 117–128 (2013).
24. Sinitshenkova, N. D. et al. The Ephemeridea (Insecta) from the Grès à Voltzia (early Middle Triassic) of the Vosges (NE France). *Paläontologische Zeitschrift* **79**, 377–397 (2005).

25. Kukalová-Peck, J. Carboniferous protodonatoid dragonfly nymphs and the synapomorphies of Odonatoptera and Ephemeroptera (Insecta: Palaeoptera). *Palaeodiversity* **2**, 169–198 (2009).

26. Huang, Diying & Nel, A. Oldest 'libelluloid' dragonfly from the Middle Jurassic of China (Odonata: Anisoptera: Cavilabiata). *Neues Jahrbuch für Geologie und Paläontologie Abhandlungen* **246,** 63–68 (2007).

27. Fleck, G. & Nel, A. Revision of the Mesozoic family Aeschnidiidae (Odonata: Anisoptera). *Zoologica* **153**, 1–180 (2003).

28. Krishna, K. et al. Treatise on the Isoptera of the world. *Bulletin of the American Museum of Natural History* **377**, 2704 pp (2013).

29. Azar, D. et al. Paramesopsocidae, a new Mesozoic psocid family (Insecta: Psocodea "Psocoptera": Psocomorpha). *Annales de la Société Entomologique de France* (N.S.) **44**, 459–470 (2009).

30. Krassilov, V. A. et al. Pollen morphotypes from the intestine of a Permian booklouse. *Review of Palaeobotany and Palynology* **106**, 89–96 (1999).

31. Nel, A. et al. Traits and evolution of wing venation pattern in paraneopteran insects. *Journal of Morphology* **273**, 480–506 (2012).

32. Nel, A. et al. The earliest-known holometabolous insects. *Nature* **503**, 257–261 (2013).

33. Krenn, H. W. et al. Mouthparts and nectar feeding of the flower visiting cricket *Glomeremus orchidophilus* (Gryllacrididae). *Arthropod Structure & Development* **45**, 221–229 (2016).

34. Nel, P. et al. From Carboniferous to Recent: wing venation enlightens evolution of thysanopteran lineage. *Journal of Systematic Palaeontology* **10**, 385–399 (2012).

35. Nel, P. et al. Redefining the Thripida (Insecta: Paraneoptera). *Journal of Systematic Palaeontology* **12**, 865–878 (2014).

36. Bekker-Migdisova, E. E. [Order Homoptera.] pp. 286-393. *In*: Rohdendorf, B. B. et al. (eds). [Paleozoic insects of the Kuznetsk basin.] *Trudy Paleontologicheskogo Instituta Akademii nauk SSSR* **85** (1961).

37. Drohojowska, J. & Szwedo, J. A new whitefly from Early Cretaceous Lebanese amber (Hemiptera: Sternorrhyncha: Aleyrodidae). *Insect Systematics & Evolution* **42**, 179–196 (2011).

38. Fraser, N. C. & Grimaldi, D. A. Who else lived in the Late Triassic? – The World of the early dinosaurs as illustrated by a fossil lagerstaette in Virginia. *Dinofest International Proceedings* **1997** 191–198 (1997).

39. Gullan, P. J. & Cook, L. G. Phylogeny and higher classification of the scale insects (Hemiptera: Sternorrhyncha: Coccoidea). *Zootaxa* **1668**, 413–425 (2007).

40. Vea, I. M. & Grimaldi, D. A. Diverse new scale insects (Hemiptera: Coccoidea) in amber from the Cretaceous and Eocene with a phylogenetic framework for fossil Coccoidea. *American Museum Novitates* **3823**, 1–15 (2015).

41. Shcherbakov, D. E. Extinct four-winged precoccids and the ancestry of scale insects and aphids (Hemiptera). *Russian Entomological Journal* **16**, 47–62 (2007).

42. Heie, O. E. & Wegierek, P. A classification of the Aphidomorpha (Hemiptera Sternorrhyncha) under consideration of the fossil taxa. *Redia* **92**, 69–77 (2009).

43. Wappler, T. et al. Scratching an ancient itch: an Eocene bird louse fossil. *Proceedings of the Royal Society of London* B (Supplement) **271**, S255–S258 (2004).

44. Haug, J. T. et al. Life habits, hox genes, and affinities of a 311 million-year-old holometabolan larva. *BMC Evolutionary Biology* **15**, 1–29 (2015).

45. Engel, M. S. A new sawfly from the Triassic of Queensland, Australia (Hymenoptera: Xyelidae), *Memoirs of the Queensland Museum* **51**, 558 (2005).

46. Zessin, W. New Upper Liassic Apocrita and the phylogeny of the Hymenoptera. *Deutsche Entomologische Zeitschrift* (N.F.) **32**, 129–142 (1985).

47. Rasnitsyn, A. P. & Zhang, Hai-chun Early Evolution of Apocrita (Insecta, Hymenoptera) as indicated by new findings in the Middle Jurassic of Daohugou, Northeast China. *Acta Geologica Sinica* English Edition **84**, 834–873 (2010).

48. Poinar, G. O. Jr. & Danforth, B. N. A fossil bee from Early Cretaceous Burmese amber. *Science* **314**, 614 (2006).

49. Ponomarenko, A. G. [Early Jurassic water beetles from the Angara river.] *Paleontologicheskij Zhurnal* **1963**, 128–131 (1963).

50. Georges, V. P. A fossil predaceous larva of an aquatic beetle Dytiscidae, from the Cretaceous limestone clay, Seminar Hills, near Takli, India. *Nagpur Current Science* **37**, 618–619 (1968).

51. Ponomarenko, A. G. Two new species of Mesozoic dysticoid beetles from Asia. *Paleontological Journal* **27**, 182-191 (1993).

52. Ghosh, S. C. et al. First record of an aquatic beetle larva (Insecta: Coleoptera) from the Parsora Formation (Permo-Triassic), India. *Palaeontology* **50**, 1335–1340 (2007).

53. Prokin, A. A. et al. New beetle larvae (Coleoptera: Coptoclavidae, Caraboidea, Polyphaga) from the Upper Triassic of Germany. *Russian Entomological Journal* **22**, 259–274 (2013).

54. Beutel, R. G. et al. On the phylogeny and evolution of Mesozoic and extant lineages of Adephaga (Coleoptera, Insecta). *Cladistics* **29**, 147–165 (2013).

55. Kazantsev, S. V. A new Luciolinae firefly (Coleoptera: Lampyridae) from the Baltic amber. *Russian Entomological Journal* **21**, 319–320 (2012).

56. Kazantsev, S. V. *Protoluciola albertalleni* gen. n., sp. n., a new Luciolinae firefly (Insecta: Coleoptera: Lampyridae) from Burmite amber. *Russian Entomological Journal* **24**, 281–283 (2015).
57. Gratshev, V. G. & Zherikhin, V. V. The fossil record of weevils and related beetle families (Coleoptera: Curculionoidea). *Acta Zoologica Cracoviensia* **46** (suppl. – Fossil Insects), 393–398 (2003).
58. Schlüter, T. Zur Systematik und Palökologie harzkonservieter Arthropoda einer Taphozönose aus dem Cenomanium von NW-Frankreich. *Berliner Geowissenschaftliche Abhandlungen, Reihe A, Geologie und Paläontologie* **9** 150 pp (1978).
59. Zhang, Xi & Zhou, Hong-Zhang How old are the rove beetles (Insecta: Coleoptera: Staphylinidae) and their lineages? Seeking an answer with DNA. *Zoological Science* **30**, 490–501 (2013).
60. Żyła, D. et al. Cretaceous origin of the unique prey-capture apparatus in megadiverse genus: stem lineage of Steninae rove beetles discovered in Burmese amber. *Nature Scientific Reports* **7** (45904), 1–15 (2017).
61. Engel, M. S. An Eocene ectoparasite of bees: The oldest definitive record of phoretic meloid triungulins (Coleoptera: Meloidae; Hymenoptera: Megachilidae). *Acta Zoologica Cracoviensia* **48B**, 43–48 (2005).
62. Poinar, G. O. Jr. & Brown, A. New genera and species of jumping ground bugs (Hemiptera: Schizopteridae) in Dominican and Burmese amber, with a description of a meloid (Coleoptera: Meloidae) triungulin on a Burmese specimen. *Annales de la Société Entomologique de France* (N.S.) **50**, 372–381 (2015).

63. Perrichot, V. et al. Two new wedge-shaped beetles in Albo-Cenomanian ambers of France (Coleoptera: Ripiphoridae: Rhipiphorinae). *European Journal of Entomology* **101**, 577–581 (2004).

64. Batelka, J. et al. New ripiphorid beetles in mid-Cretaceous amber from Myanmar (Coleoptera: Ripiphoridae): First Pelecotominae and possible Mesozoic aggregative behaviour in male Ripidiinae. *Cretaceous Research* **68**, 70–78 (2016).

65. Batelka, J. et al. Position and relationships of Ripiphoridae (Coleoptera: Tenebrionoidea) inferred from ribosomal and mitochondrial molecular markers. *Annales Zoologici* **66**, 113–123 (2016).

66. Wilhelmi, A. P. & Krenn, H. W. Elongated mouthparts of nectar-feeding Meloidae (Coleoptera). *Zoomorphology* **131**, 325–337 (2012).

67. Kirejtshuk, A. G. & Ponomarenko, A. G. A new coleopterous family Mesocinetidae fam. nov. (Coleoptera: Scirtoidea) from Late Mesozoic and notes on fossil remains from Shar-Teg (Upper Jurassic, South-Western Mongolia). *Zoosystematica Rossica* **19**, 301–325 (2010).

68. Ponomarenko, A. G. [Scarabaeiformes *incertae sedis*.] pp. 110–112. *In*: Rasnitsyn, A. P. (ed.). *Nasekomye v rannemelovykh ekosistemakh Zapadonoy Mongolii* [Insects in the Early Cretaceous Ecosystems of West Mongolia.] *Trudy Sovmestnaya Sovetsko-Mongol'skaya Paleontologicheskaya Ehkspeditsiya* **28**, 213 pp. (1986).

69. Kolibáč, J. Trogossitidae: a review of the beetle family, with a catalogue and keys. *ZooKeys* **366**, 1–194 2013).

70. Ponomarenko, A. G. et al. Upper Jurassic Lagerstätte Shar Teg, Southwestern Mongolia. Paleontological Journal **48**, 1573–1682 (2014).

71. Grimaldi, D. A. et al. Strepsiptera and Triangula in Cretaceous amber. *Insect Systematics & Evolution* **36**, 1–20 (2005).

72. Kathirithamby, J. & Engel, M. S. A revised key to the living and fossil families of Strepsiptera, with the description of a new family, Cretostylopidae. *Journal of the Kansas Entomological Society* **87**, 385–388 (2014).

73. Engel, M.S. et al. A new twisted-wing parasitoid from mid-Cretaceous amber of Myanmar (Strepsiptera). *Cretaceous Research* **58**, 160–167 (2016).

74. Krenn, H. W. et al. Flower visiting Neuroptera: mouthparts and feeding behaviour of *Nemoptera sinuata* (Nemopteridae). *European Journal of Entomology* **105**, 267–277 (2008).

75. Krenn, H. W. et al. Mouthparts of flower-visiting insects. *Arthropod Structure & Development* **34**, 1–40 (2005).

76. Martins-Neto, R. G. Remarks on the Neuropterofauna (Insecta, Neuroptera) from the Brazilian Cretaceous with keys for the identification of the known taxons. *Acta Geologica Hispanica* **35**, 97–118 (2000).

77. Malm, T. et al. The evolutionary history of Trichoptera (Insecta): a case of successful adaptation to life in freshwater. *Systematic Entomology* **38**, 459–473 (2013).

78. Ansorge, J. Revision of the "Trichoptera" described by Geinitz and Handlirsch from the Lower Toarcian of Dobbertin (Germany) based on new material. *Nova Supplementa Entomologica* **15**, 55–74 (2002).

79. Huang, Di-ying et al. A new family of moths from the Middle Jurassic (Insecta: Lepidoptera). *Acta Geologica Sinica* English Edition **84**, 874–885 (2010).

80. Sukacheva, I.D. and Aristov, D.S. 2017. New insects (Insecta: Trichoptera, Eoblattida) from the Lower Permian of Russia. *Paleontological Journal*, **51** (4): 374-381.

81. Gao, Yan et al. New genus and species of Rhyacophilidae (Insecta: Trichoptera) from the Middle Jurassic of China. *Acta Geologica Sinica* English Edition **87**, 1495–1500 (2013).

82. Wang, Mei-xia et al. New fossil caddisfly from Middle Jurassic of Daohugou, Inner Mongolia, China (Trichoptera: Philopotamidae). *Progress in Natural Science* **19**, 1427–1431 (2009).
83. Sukatsheva, I. D. New caddisflies (Trichoptera) from the Mesozoic of Middle Asia. *Paleontological Journal* **7**, 100–107 (1973).
84. Sukatsheva, I. D. & Rasnitsyn, A. P. Jurassic insects (Insecta) from the Sai-Sagul locality (Kyrgyzstan, Southern Fergana). *Paleontological Journal* **38**, 182–186 (2004).
85. Mouro, L. D. et al. Larval cases of caddisfly (Insecta: Trichoptera) affinity in Early Permian marine environments of Gondwana. *Nature Scientific Reports* **6**, 19215 (2016).
86. Kozlov, M. B. New Upper Jurassic and Early Cretaceous Lepidoptera. *Paleontological Journal* **23**, 34–39 (1989).
87. Kristensen, N. P. & Skalski, A. W. Phylogeny and Palaeontology. Chapter 2. pp. 7–25. *In*: Kristensen, N. P. (ed.). Lepidoptera, moths and butterflies. Volume 1: evolution, systematics, and biogeography. *Handbook of Zoology*, Walter de Gruyter, Berlin - New York, **4**, Arthropoda: Insecta, (35), 487 pp. (1998).
88. Labandeira, C. C. The pollination of Mid Mesozoic seed plants and the early history of long-proboscid insects. *Annals of the Missouri Botanical Garden* **97**, 469–513 (2010).
89. Sohn, Jae-Cheon et al. An annotated catalog of fossil and subfossil Lepidoptera (Insecta: Holometabola) of the world. *Zootaxa* **3286**, 1–132 (2012).
90. Riek, E. F. Four-winged Diptera from the Late Permian of Australia. *Proceedings of the Linnean Society of New South Wales* **101**, 250–255 (1977).
91. Ren, D. et al. A probable pollination mode before angiosperms: Eurasian, long-proboscid scorpionflies. *Science* **362**, 840–847 (2009).
92. Bashkuev, A. S. Nedubroviidae, a new family of Mecoptera: the first Paleozoic long-proboscid scorpionflies. *Zootaxa* **2895**, 47–57 (2011).

93. Cao, Yizi et al. Revision and two new species of *Itaphlebia* (Nannochoristidae: Mecoptera) from the Middle Jurassic of Inner Mongolia, China. *Alcheringa* **40**, 24–33 (2016).

94. Oberprieler, S. K. & Yeates, D. K. *Notoatherix antiqua* gen. et sp. nov., first fossil water snipe fly from the Late Jurassic of Australia (Diptera: Athericidae). **Zootaxa 3866**, 138–144 (2014).

95. Krzemiński, W. & Zessin, W. The Lower Jurassic Limoniidae from Grimmen (GDR). *Deutsche Entomologische Zeitschrift* (N.F.) **37,** 39–43 (1990).

96. Shcherbakov, D. Ye. et al. Triassic Diptera and initial radiation of the order. *International Journal on Diptera Research* **6**, 75–115 (1995).

97. Narchuk, E. P. A new fossil acrocerid fly from the Jurassic beds of Kazakhstan (Acroceridae). *Zoosystematica Rossica* **4**, 313–315 (1996).

98. Skuhravá, M. & Skuhravy, V. Species richness of gall midges (Diptera: Cecidomyiidae) in the main biogeographical regions of the world. *Acta Societatis Zoologicae Bohemicae* **69**, 327–372 (2006).

99. Borkent, A. The pupae of Culicomorpha - morphology and a new phylogenetic tree. *Zootaxa* **3396**, 1–98 (2012).

100. Krzemiński, W. & Krzemińska, E. Triassic Diptera: descriptions, revisions and phylogenetic relations. *Acta Zoologica Cracoviensia* **46** (suppl. – Fossil Insects), 153–184 (2003).

101. Schneeberg, K. & Beutel, R. G. The adult head structures of Tipulomorpha (Diptera, Insecta) and their phylogenetic implications. *Acta Zoologica* **92**, 316–343 (2011).

102. Beutel, R. G. & Baum, E. Longstanding entomological problem finally solved? Head morphology of *Nannochorista* (Mecoptera, Insecta) and possible phylogenetic

implications *Journal of Zoological Systematics and Evolutionary Research* **46**, 346–367 (2008).

103. McAlpine, J. F. First record of Calypterate flies in the Mesozoic era (Diptera: Calliphoridae). *The Canadian Entomologist* **102**, 342–346 (1970).

104. Rohdendorf, B. B. Novye mezozojckie nemestrinidy [New Mesozoic Nemestrinidae (Diptera).] pp. 180–189. *In*: Rohdendorf, B. B. (ed.). *Yurskie nasekomye karatau*. [*Jurassic insects of Karatau*.] Akademiya nauk SSSR Otdelenie Obshchej Biologii, Moscow: 1–252 (1968).

105. Zhang, Kui-Yan et al. New Middle Jurassic tangle-veined flies from Inner Mongolia, China. *Acta Palaeontologica Polonica* **53**, 161–164 (2008).

106. Karolyi, F. et al. Adaptations for nectar-feeding in the mouthparts of long-proboscid flies (Nemestrinidae: Prosoeca). *Biological Journal of the Linnean Society* **107**, 414–424 (2012).

107. Szucsich, N. U. & Krenn, H. W. Morphology and function of the proboscis in Bombyliidae (Diptera, Brachycera) and implications for proboscis evolution in Brachycera. *Zoomorphology* **120**, 79–90 (2000).

108. Mostovski, M. B. et al. Horseflies and athericids (Diptera: Tabanidae, Athericidae) from the Early Cretaceous of England and Transbaikalia. *Paleontological Journal* **37**, 162–169 (2003).

109. Zhang, Junfeng Snipe flies (Diptera: Rhagionidae) from the Daohugou Formation (Jurassic), Inner Mongolia, and the systematic position of related records in China. *Palaeontology* **56**, 217–228 (2013).

110. Borkent, A. & Grimaldi, D. A. The earliest fossil mosquito (Diptera: Culicidae), in Mid-Cretaceous Burmese amber. *Annals of the Entomological Society of America* **97**, 882–888 (2004).

111. Craig, D. A. A re-assessment of the systematic position of *Pseudosimulium humidus* (Westwood), an Upper Jurassic fossil dipteran. *Entomologist's Gazette* **28**, 175–179 (1977).

112. Currie, D. C. & Grimaldi, D. A. A new black fly (Diptera: Simuliidae) genus from mid Cretaceous (Turonian) amber of New Jersey. pp. 473–485. *In*: Grimaldi, D. A. (ed.). *Studies on fossils in amber, with particular reference to the Cretaceous of New Jersey*. Backhuys Publishers Leiden, 1–498 (2000).

113. Crosskey, R. W. The fossil pupa *Simulimima* and the evidence it provides for the Jurassic origin of the Simuliidae (Diptera). *Systematic Entomology* **16**, 401–406 (1991).

114. Dsouli, N. et al. Phylogenetic analyses of mitochondrial and nuclear data in haematophagous flies support the paraphyly of the genus *Stomoxys* (Diptera: Muscidae). *Infection Genetics and Evolution* **11**, 663–670 (2011).

115. Hastriter, M. W. Investigation of taxonomically important morphological features of endoparasitic bat flies of the subfamily Ascodipterinae (Diptera: Streblidae) by scanning electron microscopy. *Zootaxa* **1122**, 57–68 (2006).

116. Maa, T. Studies in Hippoboscidae (Diptera). Redescription of the fossil *Ornithomya rottensis* Statz. *Pacific Insects Monograph* **10**, 3–9 (1966).

117. Grimaldi, D. A. Vicariance biogeography, geographic extinctions, and the North American Miocene tsetse flies. pp. 178–204. *In*: Novacek, M. J. & Wheeler, Q. D. (eds). *Phylogeny and extinctions*. New York, Columbia University Press, vi + 253 pp. (1992).

118. Grimaldi, D. A. Diptera. pp. 164–186. *In*: Grimaldi, D. A. (ed.). Insects from the Santana formation, Early Cretaceous, of Brazil. *Bulletin of the American Museum of Natural History* **195**, 1–191 (1990).

119. Grimaldi, D. A. & Cumming, J. Brachyceran Diptera in Cretaceous ambers and Mesozoic diversification of the Eremoneura. *Bulletin of the American Museum of Natural History* **239**, 1–124 (1999).

120. Ren, D. et al. [*Faunae and stratigraphy of Jurassic – Cretaceous in Beijing and the adjacent areas*]. Seismic Publishing House Beijing: i–vii + 1–222. [in Chinese, with English summary.] (1995).

121. Huang, Diying et al. Diverse transitional giant fleas from the Mesozoic of China. *Nature* **483**, 201–204 (2012).
